# Generation of whole tumor cell vaccine for on-demand manipulation of immune responses against cancer under near-infrared laser irradiation

**DOI:** 10.1038/s41467-023-40207-y

**Published:** 2023-07-26

**Authors:** Jiaqi Meng, Yanlin Lv, Weier Bao, Zihui Meng, Shuang Wang, Yuanbin Wu, Shuping Li, Zhouguang Jiao, Zhiyuan Tian, Guanghui Ma, Wei Wei

**Affiliations:** 1 State Key Laboratory of Biochemical Engineering, Institute of Process Engineering, Chinese Academy of Sciences, Beijing, 100190 P. R. China; 2 School of Chemical Sciences, University of Chinese Academy of Sciences, Beijing, 100049 P. R. China; 3 Department of Hepatobiliary-Pancreatic Surgery, China-Japan Union Hospital of Jilin University, Changchun, 130033 P. R. China; 4 School of Chemical Engineering, University of Chinese Academy of Sciences, Beijing, 100049 P. R. China

**Keywords:** Cancer immunotherapy, Cell vaccines, Tumour vaccines, Nanotechnology in cancer

## Abstract

The therapeutic efficacy of whole tumor cell vaccines (TCVs) is modest, which has delayed their translation into personalized immunotherapies in the clinic. Here, we develop a TCV platform based on photothermal nanoparticle-loaded tumor cells, which can be rationally applied to diverse tumor types to achieve on-demand boost of anti-tumor immune responses for inhibiting tumor growth. During the fabrication process, mild photothermal heating by near-infrared (NIR) laser irradiation induces the nanoparticle-bearing tumor cells to express heat shock proteins as endogenous adjuvants. After a single vaccination at the back of tumor-bearing mice, non-invasive NIR laser irradiation further induces mild hyperthermia at vaccination site, which promotes the recruitment, activation, and antigen presentation by dendritic cells. Using an indicator we term fluctuation of tumor growth rate, we determine appropriate irradiation regimens (including optimized irradiation intervals and times). This TCV platform enables on-demand NIR manipulation of immune responses, and we demonstrate potent therapeutic efficacy against six murine models that mimick a range of clinical scenarios, including a model based on humanized mice and patient-derived tumor xenografts.

## Abstract (web summary)

Whole autologous tumor cell vaccine (TCV) has been proposed as a tool for cancer immunotherapy. Here the authors describe the design of a TCV platform based on photothermal nanoparticle-loaded tumor cells, triggering NIR laser irradiation induced anti-tumor immune responses at the vaccination site.

## Introduction

Tumor vaccines have long been envisioned as promising tools for cancer immunotherapies, which fight tumor cells by harnessing host immunity^1–4^. Although tumor vaccines with defined antigens have been utilized to treat cancers, the heterogeneity in antigens across patients and the high cost of personal antigen mutant identification have hindered their clinical applications^5^. To obviate these limitations, a wave of efforts has spurred the development of whole tumor cell vaccine (denoted as TCV)^6–8^, which represents a pool of all potential antigens and provides the possibility of inducing an immune response against tumor antigens specific to individual cancer^9,10^. Accordingly, an individual treated with a TCV experiences polyvalent antitumor immune responses, showing much less susceptibility to tumor escape^11^.

Owing to the poor immunogenicity of inactivated tumor cells alone, adjuvant-related approaches have been developed for TCVs^12,13^. The first known example was the use of Bacillus Calmette-Guérin (BCG) as an adjuvant mixed with inactivated hepatocellular carcinoma cells^14^. Recently, immune-stimulating molecules, such as granulocyte-macrophage colony stimulating factor (GM-CSF)^15,16^ and interleukin (IL-2)^17^, have also shown a promise in enhancing the immune responses of TCVs. To maintain the same metabolic behavior with inactivated tumor cells at the vaccination site, strategies based on directly modifying the TCVs per se have been explored, including retroviral integration to enable the tumor cells themselves to produce immune-stimulating molecules such as GM-CSF^18–20^. Although promising, the complex and time-consuming gene transfection process is challenging to perform with precious autologous tumor cells^21^.

In addition to improving immunogenicity, another challenge with TCVs is that there are currently no standardized dosing regimens. In general, multiple dosing is employed experientially to maintain an ongoing immune response, which brings several limitations for clinical use^20,22,23^. Given that autologous tumor cells are precious, such a multiple-dosage regimen raises difficulties in producing and storing TCVs sourced from patients themselves. Notably, it is not clear to what extent individual differences among patients can influence therapeutic efficacy, and the experiential regimen may not fully fit the immunotherapy requirements during the course of a malignancy^24^. Therefore, an ideal TCV should on the one hand enable simple preparation capable of endogenous adjuvant production, while on the other hand be single administration yet tunable to match the heterogenous immune responses of individual patients.

**Fig. 1:**
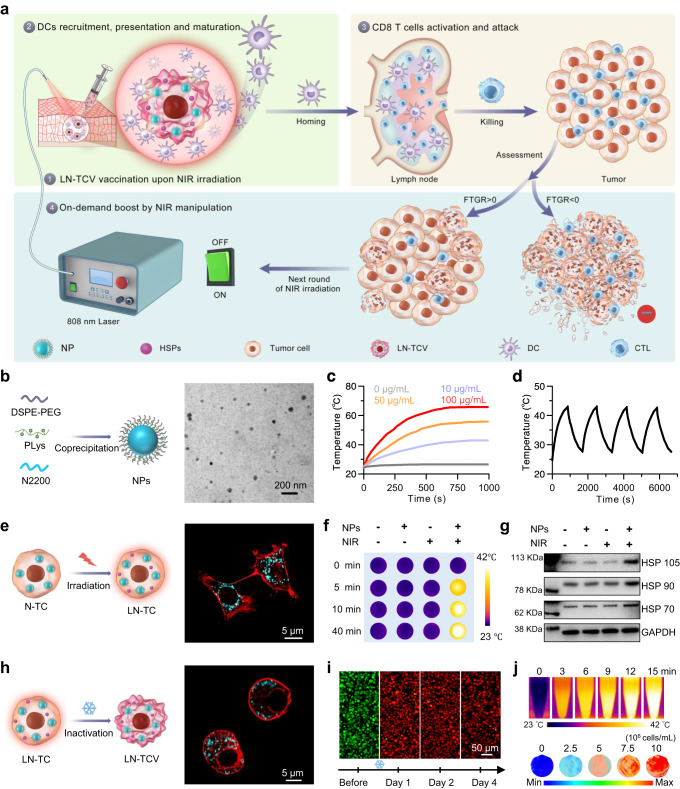
Strategy of using photothermal nanoparticle-adopted whole tumor cell vaccine (LN-TCV) for on-demand near-infrared (NIR) manipulation of immune responses against cancer and corresponding characterizations of the LN-TCV construction process. **a** Schematic illustration of NIR laser irradiation-manipulated immune responses for antitumor immunotherapy. The on-demand immune responses manipulated by NIR laser irradiation based on the fluctuation of tumor growth rate (FTGR) can promote antitumor therapeutic effects after a single vaccination. **b** Schematic illustration of the preparation (left) and transmission electron microscopy (TEM) image (right) of photothermal nanoparticles (NPs). **c** Temperature change curves for NPs with different concentrations upon continuous NIR laser irradiation (808 nm, 0.65 W/cm^2^, 1000 s). **d** Heating and cooling curves of NPs (10 μg/mL) after pulsed NIR laser irradiation (808 nm, 0.65 W/cm^2^). **e** Schematic illustration of LN-TC construction (left) and the confocal laser scanning microscopy (CLSM) image of N-TC (right). Red: Rhodamine-phalloidin-labeled-cell membrane; Cyan: P-F8-DPSB-labeled NPs. **f** NIR thermographic images of 4T1 tumor cells variously exposed to NPs and/or continuous NIR laser irradiation (808 nm, 0.65 W/cm^2^, 40 min). The cells were incubated with NPs for 12 h, and the free NPs were washed off before irradiation. **g** Western blotting analysis for the expression of HSP 70, HSP 90, and HSP 105 proteins in 4T1 tumor cells variously exposed to NPs and/or NIR laser irradiation (808 nm, 0.65 W/cm^2^, 40 min). **h** Schematic illustration of LN-TCV construction and CLSM images of LN-TCV for evaluating the cell membrane framework. Red: Rhodamine-phalloidin-labeled-cell membrane; Cyan: P-F8-DPSB-labeled NPs. **i** Live/dead analysis of LN-TCV before inactivation (before) and cultured (day 1, 2, and 4) after inactivation for demonstrating inactivated LN-TCV. Green: live cells; Red: dead cells. The images were presented with the same magnification. **j** NIR thermographic images of LN-TCV exposed to continuous NIR laser irradiation (808 nm, 0.65 W/cm^2^, 15 min) (top). Photoacoustic (PA) images of LN-TCV with different concentrations (From left to right: 0, 2.5 × 10^6^, 5 × 10^6^, 7.5 × 10^6^, and 10^7^ cells/mL) (bottom). The experiments in (**b**–**j**) were repeated three times independently with similar results. Source data were provided in the Source data file.

In this work, we design a type of TCV that supports a single injection-multiple irradiation strategy for the on-demand manipulation of local immune response (Fig. 1a). By loading photothermal nanoparticles (NPs, denoted as N) into tumor cells, the application of near-infrared (NIR) laser irradiation (denoted as L) induces the tumor cells to overexpress heat shock proteins (HSPs) as endogenous adjuvants^25^, and the resulting LN-TC is further inactivated by freeze-thaw process for obtaining the vaccine (denoted as LN-TCV). After a single vaccination at the back of tumor-bearing mice, local mild hyperthermia at vaccination site under NIR laser irradiation promotes the recruitment, activation, and presentation of dendritic cells (DCs)^26^. Upon these DCs homing to lymph nodes, T cells are specifically activated for subsequent tumor cell killing. Concurrently, we also propose an indicator for monitoring the fluctuation of tumor growth rate (FTGR), which can provide the standard for rational on-demand boost of immune response via repeated NIR laser irradiations at the vaccination site. The potent therapeutic efficacies of such an on-demand modality of TCV are demonstrated in murine triple-negative breast cancer 4T1, murine colon carcinoma CT26, murine lung carcinoma LLC, and murine pancreatic carcinoma Luc-Pan02 cells-derived tumor xenograft (CDX) models, as well as a humanized pancreatic cancer patient-derived tumor xenograft (PDX) model, highlighting the promise for personalized immunotherapy against different types of cancer.

## Results

### Fabrication and characterizations of LN-TCV

To construct LN-TCV, we first synthesized the key NPs via a coprecipitation of three components (Fig. 1b). Structurally, the photothermal copolymer N2200 (Supplementary Fig. 1a) aggregated in the hydrophobic core, whereas the amphiphilic polymer distearoyl phosphoethanolamine (DSPE)-polyethylene glycol (PEG) and poly-L-lysine (PLys) displayed on the surface. As shown in Fig. 1b and Supplementary Fig. 1b, the resulting NPs exhibited narrow size and charge distributions, with an average hydrodynamic diameter of ~50 nm and zeta potential of +28 mV. The capability of these NPs for converting NIR light to heat was then evaluated by measuring the temperature alternation of the aqueous NPs dispersion samples upon NIR laser irradiation at a substantially low output power (808 nm, 0.65 W/cm^2^). During continuous irradiation, the temperature increasing curves showed a concentration-depended manner (Fig. 1c). Taking the 10 μg/mL for example, the temperature of the solution gradually increased and reached at 43 °C. Once the irradiation was alternatively switched on and off, the solution still could be heat up to a similar level, indicating the excellent photothermal stability of the NP (Fig. 1d). Based on the heating-cooling curve, the photothermal conversion efficiency (η)^27^ of our NPs was calculated up to 73.19% (Supplementary Fig. 1c, d), considerably higher than the values of previously reported photothermal agents, such as dopamine-melanin nanoparticles (40.0%)^28^ and Cu_2_-xSi_2_O_5_(OH)_3_ (48.3%)^29^.

After ensuring the good biocompatibility of NPs (Supplementary Fig. 1e, f), we evaluated the capacities of murine triple-negative breast cancer 4T1 tumor cells to internalize the NPs and to produce mild local pyrexia upon NIR laser irradiation. Both confocal laser scanning microscopy (CLSM) and flow cytometry (FCM) analysis indicated that the tumor cells indeed internalized large amounts of NPs (Fig. 1e and Supplementary Fig. 2a), which could be attributed to the positive charge on the NPs that PLys components presented. As compared to the nearly unchanged temperature in the other counterparts without NPs and/or irradiation, the NPs-loaded tumor cells (N-TC) showed appreciable temperature elevation upon the NIR laser irradiation (Fig. 1f), with a very slight killing effect to the tumor cells (Supplementary Fig. 2b). Accordingly, the 40 min NIR laser irradiated N-TC (LN-TC) strongly upregulated the expression of HSPs, including HSP 70, HSP 90 and HSP 105 (Fig. 1g and Supplementary Fig. 2c, d), which could serve as endogenous adjuvants for DCs activation and maturation^25,30,31^. Note that neither intracellular translocation of high mobility group protein 1 (HMGB1) nor upregulated expression of calreticulin (CRT) was observed upon above condition (Supplementary Fig. 3), thus excluding the adjuvanticity of these two well-known damage-associated molecular patterns.

Before vaccination, LN-TC was inactivated by the optimized freeze-thaw process (Supplementary Fig. 4a). After two cycles, the cell membrane framework remained (Fig. 1h), and almost no change was observed for the contents of either NPs or HSPs (Supplementary Fig. 4b–e). To verify the safety of the resulting LN-TC vaccine (LN-TCV), we further cultured the inactivated sample and detected cell viability using Live/Dead assay (Fig. 1i). Supporting the very low tumorigenicity, almost all cells were detected with dead status and failed to revive even after a long culture period. Above freeze-thaw cycles exhibited negligible impact on the photothermal property of LN-TCV, since the temperature could be still elevated to 41 °C upon 10-min NIR laser irradiation (Fig. 1j). In the meantime, the adopted NPs also enabled LN-TCV to show a concentration-dependent photoacoustic (PA) signal (Fig. 1j and Supplementary Fig. 5), which gave a way to track our LN-TCV via PA imaging in vivo.

### Immune responses upon NIR laser irradiation at vaccination site

**Fig. 2:**
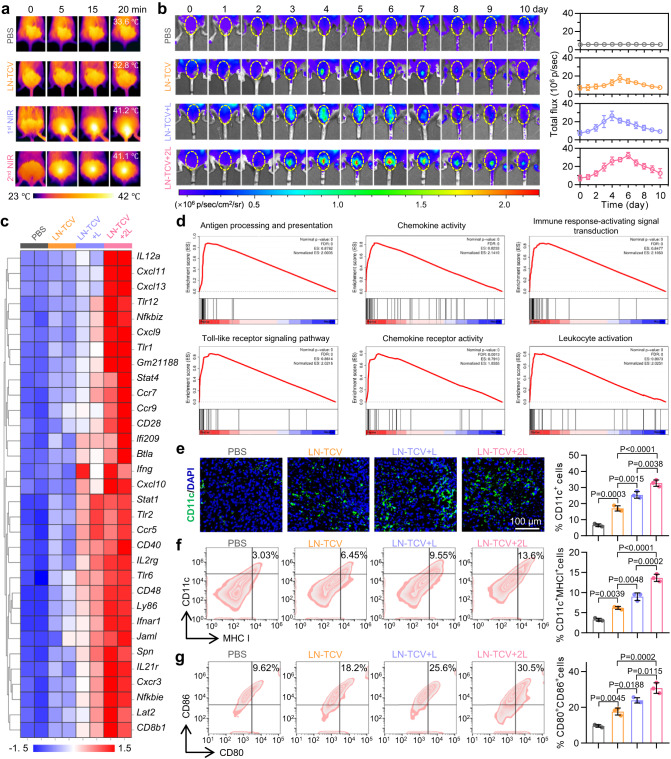
Characterizations of photothermal response and subsequent immune responses with different treatments at the vaccination site. **a** NIR thermographic images of mice in various groups (PBS, LN-TCV, LN-TCV+1^st^ NIR, and LN-TCV+2^nd^ NIR). **b** Representative bioluminescence images (left) and quantitative analysis (right) of IFN-γ-IRES-Venus-AkaLuc mice in different groups (PBS, LN-TCV, LN-TCV + L, and LN-TCV + 2L). **c** Transcriptome analysis of the vaccination sites (back of the mice) with indicated treatments at day 6. Differential gene cluster analysis was shown as a heat map (*n* = 2 mice per group). **d** Gene set enrichment analysis (GSEA) for the altered gene sets in the LN-TCV + 2L treatment group versus the PBS group. **e** Immunofluorescence slices (left) and quantitative analysis (right) of DCs (CD11c^+^) at the vaccination sites (back of the mice) from indicated groups at day 6. Green: CD11c^+^cells; Blue: cell nucleus. The images were presented with the same magnification. The *P* values of LN-TCV to PBS, LN-TCV + L to LN-TCV, LN-TCV + 2L to LN-TCV + L, and LN-TCV + 2L to LN-TCV were 0.0003, 0.0015, 0.0038, and <0.0001, respectively. **f** Representative flow cytometry plots (left) and quantitative analysis (right) of CD11c^+^MHC I^+^ cells at the vaccination sites (back of the mice) from indicated groups at day 6. The *P* values of LN-TCV to PBS, LN-TCV + L to LN-TCV, LN-TCV + 2L to LN-TCV + L, and LN-TCV + 2L to LN-TCV were 0.0039, 0.0048, 0.0002, and <0.0001, respectively. **g** Representative flow cytometry plots (left) and quantitative analysis (right) of mature DCs (CD80^+^CD86^+^ in CD11c^+^ gate) at the vaccination sites (back of the mice) from indicated groups at day 6. The *P* values of LN-TCV to PBS, LN-TCV + L to LN-TCV, LN-TCV + 2L to LN-TCV + L, and LN-TCV + 2L to LN-TCV were 0.0045, 0.0188, 0.0115, and 0.0002, respectively. Quantitative data in (**b**), (**e**), (**f**), and (**g**) were represented as mean values ± s.d., *n* = 3 mice per group. *P* values in (**e**), (**f**), and (**g**) were calculated by one-way ANOVA. The images and flow cytometry plots in (**a**), (**b**), (**e**), (**f**), and (**g**) were representative of three mice. Source data were provided in the Source data file.

Considering the good photothermal properties of LN-TCV in vitro, we envisioned that the NIR laser irradiation at the vaccination site could induce mild hyperthermia in vivo, forming a mild inflammatory environment locally for improving the immune responses. Pursuing this, we started our in vivo experiments with the monitor of thermogenic effect at the vaccination site (Fig. 2a). In both phosphate-buffered saline (PBS) and LN-TCV groups, a stable temperature around 33 °C was observed at the vaccination site. Once the NIR laser irradiation was executed, the temperature quickly rose to 41 °C and maintained. Owing to the long retention time of LN-TCV at the vaccination site (Supplementary Fig. 6a), a much similar mild hyperthermia was observed upon a repeated irradiation, shedding a light on the on-demand NIR manipulation.

To investigate whether mild hyperthermia generated by irradiated LN-TCV could effectively induce, as designed, a local inflammatory environment, IFN-γ-IRES-Venus-AkaLuciferase mice were used to visualize IFN-γ production based on bioluminescence imaging at the vaccination site (Fig. 2b). In addition to the PBS group, the rest of mice receiving a single vaccination at the back were randomly divided into three groups: an LN-TCV group without any NIR laser irradiation, an LN-TCV + L group given 20 min NIR laser irradiation immediately after vaccination at day 0, and an LN-TCV + 2L group given a second 20 min NIR laser irradiation at day 5. Compared to the PBS group without any IFN-γ signal, the LN-TCV group showed a slow and slight increase of IFN-γ production, which was associated with endogenous HSPs adjuvants, and this signal started to fade away at day 6. In sharp contrast, the executed irradiation after vaccination elicited a rapid and robust IFN-γ production with a peak at day 4, whereas the group given another irradiation at day 5 showed a further increase of IFN-γ production, leading to the signal peaking at day 6 and lasting for longer time. Note that no signs of redness and swelling were observed at the vaccination site (Supplementary Fig. 6b), indicating a mild and acceptable inflammatory environment.

Above distinct IFN-γ production prompted us to gain a deep insight into the underlying inflammatory mechanism. To this end, we conducted RNA sequencing to identify differentially expressed genes in the transcriptomes of BALB/c mice at day 6 after the same treatments. The vaccination sites indeed showed an upregulated expression of many immune activation-related genes, such as CCR5, CXCL10, STAT1, CD40, and TLR2, with the extent increasing in the sequence of LN-TCV, LN-TCV + L, and LN-TCV + 2L (Fig. 2c). Moreover, gene set enrichment analysis (GSEA) based on the LN-TCV + 2L group versus the PBS group revealed that GO gene sets in antigen processing and presentation, chemokine activity, immune response-activating signal transduction, toll-like receptor signaling pathway, chemokine receptor activity, and leukocyte activation were significantly upregulated (Fig. 2d).

Given that the GSEA data highly correlated with antigen-presenting cells (APCs), we next focused our attention on the impact of local inflammatory environment on DCs, a most efficient type of APCs. Accordingly, we used immunofluorescence and FCM to analyze the DCs recruitment and maturation at the vaccination site (Fig. 2e–g and Supplementary Fig. 6c, d). Compared to PBS group, vaccination with LN-TCV group showed higher levels of DCs recruitment (indicated by the number of CD11c^+^ cells) and maturation (indicated by the percentages of CD11c^+^MHC I^+^ and CD80^+^CD86^+^ cells), owing to the improved tumor antigen uptake mediated by NPs (Supplementary Fig. 7) and the adjuvant effect of endogenous HSPs released from LN-TCV (Supplementary Figs. 8, 9). The levels of these DCs indicators continuously increased upon NIR laser irradiation and further increased upon second irradiation. Collectively, these results established that LN-TCV was retained at vaccination site and that NIR laser irradiation induced mild hyperthermia to form a local inflammatory environment. Upon this, an increased number of DCs were recruited at vaccination site, facilitating the uptake and presentation of antigens released from LN-TCV.

### DCs homing and T cell activation in lymph node

**Fig. 3:**
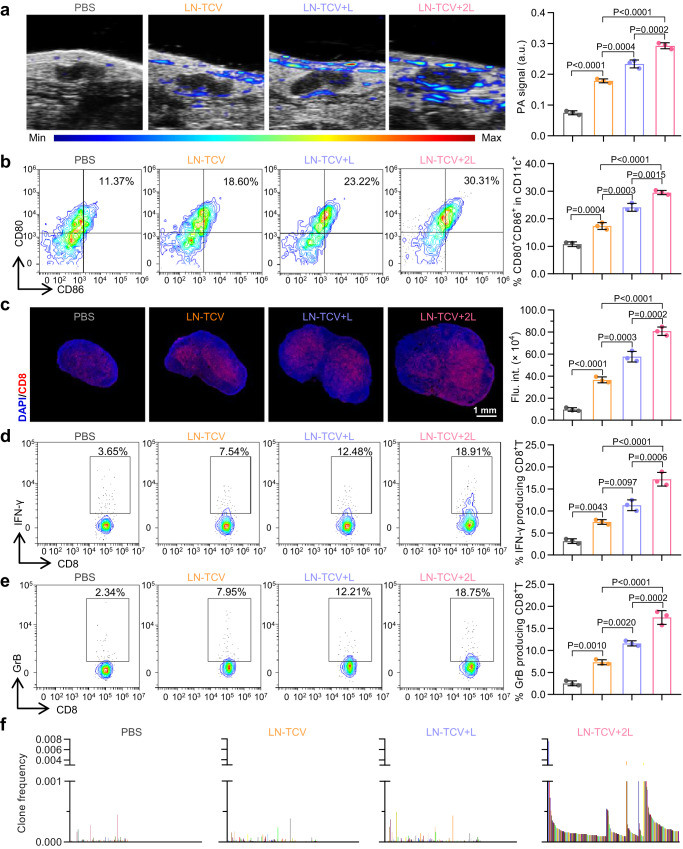
Immune responses activated by LN-TCV with NIR laser irradiation at post-injection day 9 in the lymph node. **a** PA images (left) and corresponding PA signal quantitative analysis (right) around the inguinal lymph nodes of mice with different treatments (PBS, LN-TCV, LN-TCV + L, and LN-TCV + 2L) for showing the homing of DCs. The *P* values of LN-TCV to PBS, LN-TCV + L to LN-TCV, LN-TCV + 2L to LN-TCV + L, and LN-TCV + 2L to LN-TCV were <0.0001, 0.0004, 0.0002, and <0.0001, respectively. **b** Representative flow cytometry plots (left) and quantitative analysis (right) of mature DCs (CD80^+^CD86^+^ in CD11c^+^ gate) in the lymph nodes of mice with indicated treatments. The *P* values of LN-TCV to PBS, LN-TCV + L to LN-TCV, LN-TCV + 2L to LN-TCV + L, and LN-TCV + 2L to LN-TCV were 0.0004, 0.0003, 0.0015, and <0.0001, respectively. **c** Immunofluorescence analysis (left) and quantitative analysis (right) of CD8^+^ T cells in the lymph nodes of mice with indicated treatments. Red: CD8^+^T cells; Blue: cell nucleus. The images were presented with the same magnification. The *P* values of LN-TCV to PBS, LN-TCV + L to LN-TCV, LN-TCV + 2L to LN-TCV + L, and LN-TCV + 2L to LN-TCV were <0.0001, 0.0003, 0.0002, and <0.0001, respectively. **d** Representative flow cytometry plots (left) and quantitative analysis (right) of IFN-γ-producing CD8^+^ T cells in the lymph nodes of mice with indicated treatments. The *P* values of LN-TCV to PBS, LN-TCV + L to LN-TCV, LN-TCV + 2L to LN-TCV + L, and LN-TCV + 2L to LN-TCV were 0.0043, 0.0097, 0.0006, and <0.0001, respectively. **e** Representative flow cytometry plots (left) and quantitative analysis (right) of granzyme B-producing CD8^+^ T cells in the lymph nodes of mice with indicated treatments. The *P* values of LN-TCV to PBS, LN-TCV + L to LN-TCV, LN-TCV + 2L to LN-TCV + L, and LN-TCV + 2L to LN-TCV were 0.0010, 0.0020, 0.0002, and <0.0001, respectively. **f** Clone frequencies of CDR3 sequences in the T cells derived from the lymph nodes of mice with indicated treatments (*n* = 1 mice per group). Quantitative data in (**a**–**e**) were represented as mean values ± s.d., *n* = 3 mice per group. *P* values in (**a**–**e**) were calculated by one-way ANOVA. The images and flow cytometry plots in (**a**–**e**) were representative of three mice. Source data were provided in the Source data file.

Having demonstrated the proof-of-concept for using a non-invasive NIR manipulation to fine-tune the local immune responses of LN-TCV at the vaccination site, we subsequently investigated whether the maturated DCs could homing to lymph nodes and further activate T cells. Given that the NPs in LN-TCV were internalized by DCs in vivo, we could utilize the photoacoustic properties of NPs to track the DCs homing process. As shown in Fig. 3a, the PA signals around the inguinal lymph nodes at day 9 increased in the sequence of PBS, LN-TCV, LN-TCV + L, and LN-TCV + 2L. As a result, LN-TCV + 2L group showed the highest numbers of mature DCs in the inguinal lymph nodes (Fig. 3b and Supplementary Fig. 10).

Immunofluorescence analysis of lymph node sections further supported that the increasing trend of CD8^+^T cells signal still follow the sequence: PBS < LN-TCV < LN-TCV + L < LN-TCV + 2L (Fig. 3c), indicating that LN-TCV + 2L induced the most proliferation of CD8^+^ T cells (Supplementary Fig. 11). Moreover, FCM assessing levels of IFN-γ- and granzyme B-producing CD8^+^ T cells enabled to yield a quantitative assessment of CD8^+^ T cell activity, and the LN-TCV + 2L group again outperformed other counterparts (Fig. 3d, e and Supplementary Fig. 12). As LN-TCV provided a pool of multiple tumor antigens, these stimulated T cells showed richer clonal diversity than those in the PBS control group (Fig. 3f). As the number of NIR laser irradiations increased, the clonal diversity and clone frequency of CDR3 sequences increased. Note that above the data sourced from four groups could be clearly represented as four corresponding clusters, unequivocally showing the substantial discrepancy between different treatments (Supplementary Fig. 13). Collectively, these results supported that the most efficient DCs homing in the LN-TCV + 2L mice resulted in the greatest extent of CD8^+^ T cell proliferation, activity, and multi-antigenic response.

### LN-TCV-based immunotherapy against 4T1 tumor

**Fig. 4:**
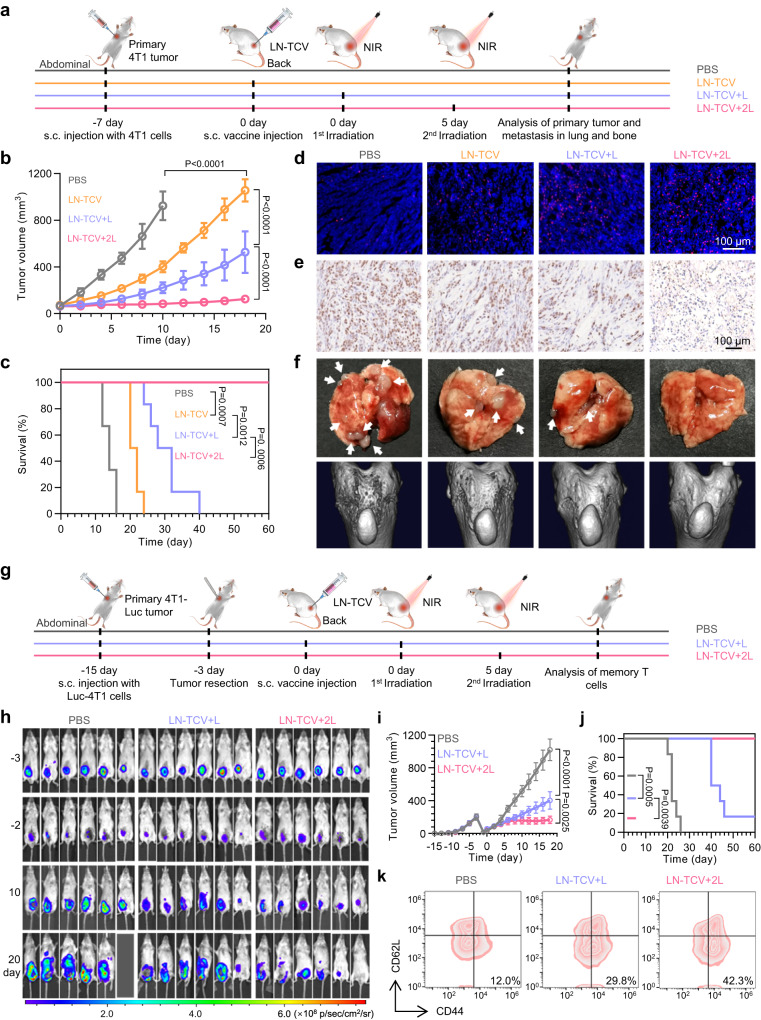
Immune responses activated by LN-TCV with NIR laser irradiation against 4T1 tumor models. **a** Experimental design for evaluating the therapeutic effects on 4T1 model. **b** Average tumor growth curves in different groups (PBS, LN-TCV, LN-TCV + L, and LN-TCV + 2L). The *P* values of LN-TCV to PBS, LN-TCV + L to LN-TCV, and LN-TCV + 2L to LN-TCV + L were all <0.0001. **c** Overall survival curves of the tumor-bearing mice in indicated groups. The *P* values of LN-TCV to PBS, LN-TCV + L to LN-TCV, and LN-TCV + 2L to LN-TCV + L were 0.0007, 0.0012, and 0.0006, respectively. **d** Immunofluorescence analysis of CD8^+^ T cells in tumors. Red: CD8^+^ T cell; Blue: cell nucleus. The images were presented with the same magnification. **e** Representative sections for cell proliferation analysis of tumor tissue by using Ki67 staining. Brown: proliferated cells. The images were presented with the same magnification. **f** Representative images of gross morphology of lung tissues (top) and computed tomography (CT) images of spontaneous tibia metastasis (bottom) in indicated groups. White arrow: lung metastasis. **g** Experimental design for evaluating the therapeutic effects on the postoperative Luc-4T1 model. **h** In vivo bioluminescence images of mice in indicated groups on various days (*n* = 6 mice per group). **i** Average tumor growth curves in different groups (PBS, LN-TCV + L, and LN-TCV + 2L). The *P* values of LN-TCV + L to PBS and LN-TCV + 2L to LN-TCV + L were <0.0001 and 0.0025. **j** Overall survival curves of the tumor-bearing mice in indicated groups. The *P* values of LN-TCV + L to PBS and LN-TCV + 2L to LN-TCV + L were 0.0005 and 0.0039. **k** Representative flow cytometry plots of effector memory T cells in the spleen examined in indicated groups at day 20. Quantitative data in (**b**) and (**i**) were represented as mean values ± s.d., *n* = 2 independent experiment, *n* = 6 mice per group. The experiments in (**c**) and (**j**) were *n* = 6 mice per group. *P* values in (**b**) and (**i**) were calculated by one-way ANOVA. *P* values in (**c**) and (**j**) were calculated by log-rank test. The images in (**d**–**f**) were representative of six mice, and the flow cytometry plots in (**k**) were representative of three mice. Source data were provided in the Source data file.

Above results motivated us to investigate in vivo therapeutic performance of our NIR-responsive vaccine. For comparison, 4T1 tumor-bearing BALB/c mice were randomly grouped as described earlier (Fig. 4a). Compared with the PBS group, LN-TCV exerted modest tumor growth inhibition. Moreover, the increasing numbers of NIR laser irradiation unequivocally improved the inhibition of tumors, with the tumor size diminishing in the sequence of LN-TCV, LN-TCV + L, and LN-TCV + 2L (Fig. 4b), which was consistent with previous data showing similar trends in the immune responses of DCs (Fig. 3b) and CD8^+^ T cells (Fig. 3c–e). Consequently, treating tumor-bearing mice with LN-TCV + 2L nearly completely inhibited tumor growth with a 100% survival rate after 60 days (Fig. 4c), outperforming that in the groups of TCV + 2L, TCV + BCG^14,32^, N-TCV + BCG + 2L, and N-TCV + 2L (Supplementary Figs. 14, 15). Such a potent therapeutic effect in LN-TCV + 2L was further supported by the most prominent infiltration of CD8^+^ T cells in tumor and the most efficient suppression of tumor proliferation (Fig. 4d, e and Supplementary Fig. 16a, b).

Considering the highly metastatic property of 4T1 tumors, we also conducted experiments to assess the impact of cancer vaccination on both lung and bone metastases (Fig. 4f and Supplementary Fig. 16c, d). Examining the same four experimental groups and counting the number of metastatic foci in the lungs of mice sacrificed at day 10 revealed no obvious metastasis in the LN-TCV + 2L group, while the number of metastatic foci in the other groups reflected similar trends as the above data. Likewise, micro-computed tomography analysis conducted at day 14 showed no metastasis-induced erosion in the tibia of LN-TCV + 2L mice, but increasing extents of erosion in the LN-TCV + L, LN-TCV, and PBS groups. Note that the potent therapeutic outcomes in LN-TCV + 2L were achieved with few abnormalities of haematoxylin and eosin (H&E)-stained micrographs and serum biochemistry data (Supplementary Fig. 16e, f), indicating the safety of a single LN-TCV vaccination combined with repeated irradiations.

As a common clinical problem, residual tumor cells missed by surgical excision frequently resulted in tumor recurrence. To address this issue, we investigated the applicability for preventing postsurgical recurrence. To this end, we established a recurrence model by surgically resecting most of the Luc-4T1 primary tumor (Fig. 4g). Specifically, confirming the proper residual cancer cells burden for this recurrence model, we detected no differences in bioluminescence intensity among the three experimental groups of mice (PBS, LN-TCV + L, and LN-TCV + 2L) after tumor resection (Fig. 4h). After vaccination at back of tumor-bearing mice, growth of the recurrent tumors was significantly inhibited in both groups that received NIR laser irradiation, with a greater extent of inhibition observed in the LN-TCV + 2L group (Fig. 4i). Accordingly, none of the LN-TCV + 2L group mice died within 60 days, whereas all of the PBS group mice had died within 26 days (Fig. 4j). Such a satisfactory potency was found highly correlated with immune memory, as effector memory T cells, showed a significant increase in the mice treated with LN-TCV + 2L (Fig. 4k and Supplementary Fig. 17). These results again highlighted the impact of NIR laser irradiation on increasing the antitumor effect of vaccination.

### On-demand NIR manipulation of LN-TCV for various tumor models

All experiments up to this point were based on 4T1 cells, including in vitro vaccine construction prior to vaccination, evaluations of immune responses, and therapeutic impacts in xenograft models. Next, we determined whether this NPs-adopted and photothermal tunable TCV platform was suitable for other types of tumors. First of all, the same protocols were used for in vitro vaccine construction using various tumor cells prior to vaccination and subsequent antitumor therapy. As shown in Supplementary Fig. 18, CLSM images again confirmed that two freeze-thaw cycles didn’t disrupt the cell membrane framework of LN-TCVs sourced from murine colon carcinoma CT26 cells, murine Lewis lung carcinoma LLC cells, or murine pancreatic carcinoma Luc-Pan02 cells. Meanwhile, the western blotting assay proved that all three LN-TCVs containing higher expression levels of HSPs after treating with NIR laser irradiation (Supplementary Fig. 18). In addition, they also possessed excellent photothermal properties, further supporting the successful construction of LN-TCVs based on the other three types of tumor cells.

**Fig. 5:**
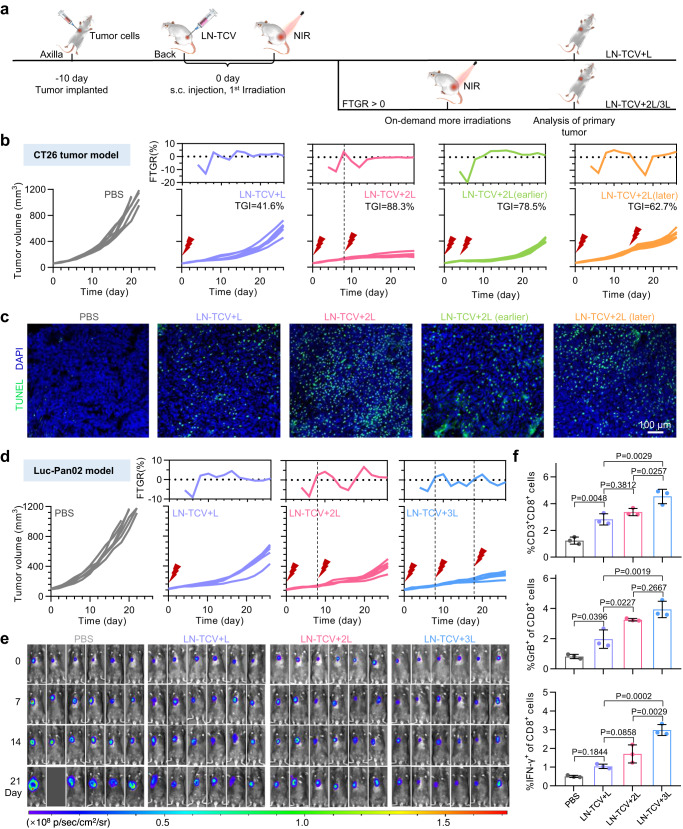
Universality of LN-TCV manipulated by on-demand NIR laser irradiation for inhibiting diverse tumor models. **a** Experimental design to evaluate tumor inhibition in CT26 and Luc-Pan02 tumor models. **b** CT26 tumor growth curves (bottom) and the corresponding FTGR values (top) analysis in different groups (PBS, LN-TCV + L, LN-TCV + 2L, LN-TCV + 2L(earlier), and LN-TCV + 2L(later)). Mice in the LN-TCV + 2L group received the second NIR laser irradiation (808 nm, 0.65 W/cm^2^, 20 min) at day 8 according to the value of FTGR. However, mice in the LN-TCV + 2L(earlier) and the LN-TCV + 2L(later) received second NIR laser irradiation (808 nm, 0.65 W/cm^2^, 20 min) at earlier (day 4) or later (day 14), respectively. The tumor growth inhibition (TGI) in each treatment group was calculated at day 20. **c** TUNEL analysis in CT26 tumors with indicated treatments. **d** Luc-Pan02 tumor growth curves (bottom) and corresponding FTGR values (top) analysis in indicated groups. According to the value of FTGR, mice in LN-TCV + 2L received the second NIR laser irradiation (808 nm, 0.65 W/cm^2^, 20 min) at day 8 and mice in LN-TCV + 3L received the third NIR laser irradiation (808 nm, 0.65 W/cm^2^, 20 min) at day 18. **e** In vivo bioluminescence images of the Luc-Pan02 tumor-bearing mice in different groups (PBS, LN-TCV + L, LN-TCV + 2L, and LN-TCV + 3L) at various days. **f** Quantitative analysis of tumor-infiltrating CD8^+^ T cells and phenotyping with infiltrated CD8^+^ T cells in tumors of indicated groups. For the percentage of CD3^+^CD8^+^cells, The *P* values of LN-TCV + L to PBS, LN-TCV + 2L to LN-TCV + L, LN-TCV + 3L to LN-TCV + 2L, and LN-TCV + 3L to LN-TCV + L were 0.0048, 0.3812, 0.0257, and 0.0029, respectively. Quantitative data in (**f**) were represented as mean values ± s.d., *n* = 3 mice per group. *P* values in (**f**) were calculated by one-way ANOVA. The experiments in (**b**), (**d**), and (**e**) were repeated two times independently with similar results, *n* = 6 mice per group. The images in (**c**) were representative of three mice. Source data were provided in the Source data file.

Given that different types of tumors have their own features of development and sensitivity to immunotherapy, we were interested in the applicability of executing repeated NIR laser irradiation with rationally appropriate times and intervals after the LN-TCV vaccination and first irradiation (Fig. 5a). Such an on-demand NIR manipulation strategy could fully fit the immunotherapy requirements during the course of a malignancy but required a reliable indicator to help us estimate rational irradiation regimen. Inspired by tumor growth rate (TGR) used to evaluate the effect of tumor inhibition in clinic^33^, we proposed an indicator, denoted FTGR. Specifically, the value of FTGR < 0 meant that the TGR declined, indicating a good susceptibility to the existing immune response. In contrast, the rise of TGR led to the value of FTGR > 0, providing a sign of requiring boosted immune response.

Building on our proposed FTGR indicator and LN-TCV with excellent maneuverability conferred by NIR laser irradiation, we next investigated the feasibility of making customized irradiation regimen for various types of tumors. In the experiment of CT26 tumor model, we prepared CT26 tumor-bearing BALB/c mice for receiving LN-TCV vaccination and the first irradiation at day 0. Compared to the PBS group, these mice after LN-TCV + L treatment indeed showed inhibited tumor growth. After calculating the FTGR, we found that the value in the beginning gradually declined, and reached to −13.2% at day 6. However, this value rallied over 0 at day 8, hinting at the subsequent rapid tumor growth and reminding us to execute the second irradiation at this time point. As excessive number of tumor-bearing mice had been prepared for the first irradiation, we next randomly selected six mice to monitor the subsequent tumor development without further treatment, while the other mice underwent a second irradiation. Owing to the resulting boosted immune responses, the tumor development of mice receiving LN-TCV + 2L treatment was well inhibited (Fig. 5b, c). All the mice in LN-TCV + L group died within day 40, while the LN-TCV + 2L group showed a 100% survival rate at day 60 (Supplementary Fig. 19a). Note that the second irradiation executed at impropriate time points (earlier at day 4 or later at day 14) significantly compromised the tumor growth inhibition rate, thus emphasizing rationality of utilizing FTGR value for executing on-demand NIR irradiation.

Extending the tumor type to lung cancer, we utilized the FTGR value to rationally execute the second irradiation at day 10 in the LLC murine lung cancer model, which significantly improved tumor inhibition and survival rate (Supplementary Fig. 19b–d). In addition, we also tested another tumor type, pancreatic carcinoma, one of the most lethal cancers worldwide^34^. After establishing the Luc-Pan02 pancreatic carcinoma model, we immunized the excessive mice with LN-TCV and executed the first irradiation at day 0. When the FTGR value exceeding 0 at day 8, we selected six mice for monitoring subsequent tumor development without further treatment, while the other mice underwent a second irradiation. Owing to the malignancy of this tumor, we observed that FTGR value again exceeded 0 at day 18, which prompted us to repeat the random selection of mice already receiving LN-TCV + 2L treatment for monitoring the subsequent tumor development, while other mice underwent a third irradiation. As a result, the tumor development was well inhibited in the LN-TCV + 3L group (Fig. 5d), which was also supported by the in vivo imaging of the tumor bioluminescence signal (Fig. 5e) as well as the 100% survival rate during 60 days (Supplementary Fig. 19e).

Given that the most potent therapeutic effect was achieved in the LN-TCV + 3L group (Supplementary Fig. 19f), we were also interested in the potential impacts of such multiple irradiations. Considering the essential role of CD8^+^ T cells in tumor inhibition (Supplementary Fig. 20), we herein focused on their features after infiltrating into tumors. As shown in Fig. 5f, both their amounts and activities increased with the irradiation times, thus recalling the excellent maneuverability conferred by NIR irradiation for improved therapeutic outcomes. Moreover, we showed that using the LN-TCV produced from 4T1 cells to treat the Luc-Pan02 tumor model substantially hampered the therapeutic efficacy, demonstrating the specificity of the immune response (Supplementary Fig. 21). In addition, even three irradiations resulted in few abnormalities (Supplementary Fig. 22) on H&E-stained micrographs and serum biochemistry data, further indicating the safety of using multiple on-demand NIR irradiation to manipulate the immune response against cancer.

### Construction of LN-TCV from patient-derived tumor cells and therapeutic efficacy of on-demand NIR manipulation in a humanized PDX model

**Fig. 6:**
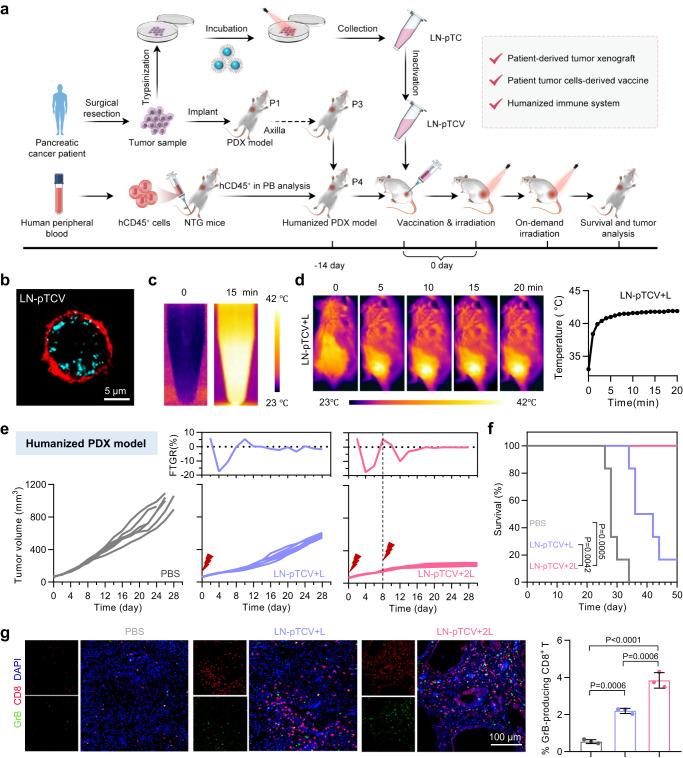
Characterizations of patient tumor cells-derived LN-pTCV and corresponding on-demand NIR laser irradiation for inhibiting a humanized pancreatic cancer PDX model. **a** Experimental design and LN-pTCV construction for evaluating antitumor effect in a humanized pancreatic cancer PDX model. PDX tissue was subcutaneously inoculated at the axilla of mice. LN-pTCV was subcutaneously injected at the back of the mice, and the NIR laser irradiation was executed at the same site. **b** CLSM image of patient tumor cell-derived LN-pTCV. Red: cell membrane; Cyan: NPs. **c** NIR thermographic images of patient tumor cell-derived LN-pTCV upon NIR laser irradiation (808 nm, 0.65 W/cm^2^, 15 min). **d** NIR thermographic images (left) and quantitative analysis (right) of mice treated with NIR laser irradiation (808 nm, 0.65 W/cm^2^, 20 min). **e** PDX tumor growth curves (bottom) and corresponding FTGR values analysis (top) in different groups (PBS, LN-pTCV+L, and LN-pTCV+2L). **f** Overall survival curves in indicated groups. The *P* values of LN-pTCV+2L to PBS and LN-pTCV+2L to LN-pTCV+L were 0.0005 and 0.0042. **g** Immunofluorescence analysis (left) and quantitative analysis (right) of granzyme B-producing CD8^+^ T cells in tumors. Red: CD8^+^ T; Green: granzyme B; Blue: cell nucleus. The images were representative of three mice and were presented with the same magnification. The *P* values of LN-pTCV+2L to PBS, LN-pTCV+L to PBS, and LN-pTCV+2L to LN-pTCV+L were <0.0001, 0.0006, and 0.0006, respectively. Quantitative data in (**g**) were represented as mean values ± s.d., *n* = 3 mice per group. *P* values in (**g**) were calculated by one-way ANOVA. *P* values in (**f**) were calculated by log-rank test. The experiments in (**e**) and (**f**) were *n* = 6 mice per group. The experiments in (**b**), (**c**), and (**d**) were repeated three times independently with similar results. Source data were provided in the Source data file.

To further confirm the clinical applicability of our platform, we next constructed patient-derived LN-TCV (LN-pTCV) and evaluated the therapeutic efficacy of on-demand NIR manipulation in a matched humanized PDX model^35^ (Fig. 6a). Initially, a tumor sample resected from a patient with pancreatic cancer was utilized for harvesting the tumor cells and following the same method to construct a personalized LN-pTCV. The internalization of NPs, the increased levels of HSPs expression, and the preserved cell membrane framework together voted the successful construction of LN-pTCV from patient-derived tumor cells (Fig. 6b and Supplementary Fig. 23a). Moreover, thermal images confirmed the photothermal property of LN-pTCV in vitro and in vivo after NIR laser irradiation (Fig. 6c, d), paving the way for subsequent on-demand NIR manipulation. To construct the humanized PDX model, tumor masses from the above resected tumor sample were transplanted into the axilla of NOD-Prkd^em26^Il2rg^em26^/Nju (NTG) mice. After engraftment for three passages, HLA-matched human peripheral blood mononuclear cells (PBMCs) were transferred into tumor-bearing NTG mice to reconstruct the human immune system (Supplementary Fig. 23b–d and Supplementary Fig. 24). After another two weeks, the resulting humanized PDX mice could be vaccinated with LN-pTCV to evaluate therapeutic benefits from the on-demand NIR manipulation.

Compared to the PBS group, the first irradiation (LN-pTCV+L) indeed resulted in a decline in FTGR value, with a minimum of −17.35% at day 4. Subsequently, the FTGR value rallied and exceeded 0 at day 8. Accordingly, we randomly selected six mice for monitoring tumor development, while the other mice underwent a second irradiation. As a result, the tumor growth in LN-pTCV+2L was well inhibited, leading to all mice remaining alive at day 50 (Fig. 6e, f). On the contrary, mice in the LN-pTCV+L group still suffered from a continuously increasing tumor burden, with only one mouse survived at day 44. Besides, this on-demand treatment (NIR laser irradiation) resulted in a further improvement on both number and activity of tumor-infiltrating CD8^+^ T cells (Fig. 6g). Collectively, these encouraging results based on personalized vaccine and humanized PDX models further strengthened the promise of our LN-pTCV combining with on-demand NIR manipulation for personalized immunotherapy against cancer.

## Discussion

In summary, we have developed a type of TCV (LN-TCV) by loading photothermal NPs into tumor cells, which supported a single injection-on-demand irradiation strategy. The preparation of LN-TCV enabled the generation of HSPs as endogenous adjuvants via a gentle and easy-doing irradiation. After a single vaccination, NIR laser irradiation induced mild hyperthermia at vaccination site to form inflammatory environment locally, which resulted in an improved immune response at the vaccination site and subsequently in lymph nodes. Notably, we proposed an FTGR indicator, which helped us estimate the irradiation regimen with the rational times and intervals. This on-demand NIR manipulation could fulfill the immunotherapy requirements for various tumors with different malignancies, which had been demonstrated across six murine models that not only mimicked a range of clinical requirements but also included a model based on humanized mouse and patient-derived tumor xenograft.

Our proof-of-concept design opens up a way of exploring optically manipulating immune responses for on-demand antitumor therapeutics with enhanced precision and anticancer efficacy. In addition to tumor cells, the tumor microenvironment also contains various stromal cells^36^, such as tumor-associated macrophages, and cancer-associated fibroblast, among others. Thus, it may be possible to extend our NPs-adopted and NIR-tunable vaccine concept from tumor cells to mixed cells from tumor tissue^37^. This may provide an antigen pool containing richer antigens, raising the possibility of eliciting a more profound immune response to both tumor cells and stroma cells, and a further improved therapeutic efficacy can be expected.

Although the long wavelength of NIR laser has the advantage of less energy dissipation in vivo, the depth of penetration (<10 mm) still fails to reach most tumor localizing in deep tissues, limiting its applications for traditional photothermal therapy against cancer. In sharp contrast, vaccination with a depth range of 2.5-10 mm is relatively shallow. In this case, superficial irradiation with NIR laser ensures the photons penetrating the skin, arriving the site of injected LN-TCV, and exciting the NPs to generate mild hyperthermia, which further induces local inflammation for boosting immune response. In addition, we can also expect that our strategy of applying local mild hyperthermia to enhance immune response can be deployed beyond TCV in theory, for example to boost the therapeutic efficacy of vaccines based on the identified neoantigens.

The future direction of our study is extending the clinical applicability and scenario. In the present work, only NIR laser was employed for producing endogenous adjuvant and boosting immune response. We envision that the utilization of laser irradiation may require specific considerations during clinical translation. For example, the very narrow linewidth of laser should match the optimum wavelength of photothermal agents, leading to customized laser source and restricted photothermal agents. Moreover, the high cost and poor portability of laser equipment may also compromise the intention of using this strategy for both patients and clinicians. In the future, perhaps the laser-based heating used in the present study can be replaced by a light-emitting diode (LED), such as photoluminescent (PL)-LED with broadband wavelength, low cost, and high brightness. Remote-controlled and wearable patch LED can also be designed, which can further improve the compliance for patients and the maneuverability for clinicians. We can even expect a scenario where patients vaccinated with TCV are treated by telemedicine in the comfort of their homes with the aid of their smartphones according to the instruction from clinicians, achieving effectively point-of-care manipulation of immune response and personalized medicine.

## Methods

### Study approval

This study was performed in strict accordance with the Regulations for the Care and Use of Laboratory Animals and Guideline for Ethical Review of Animal (China, GB/T 35892-2018). All animal experiments were reviewed and approved by the Animal Ethics Committee of the Institute of Process Engineering (approval ID: IPELLSC032101). PDX studies were approved by the Scientific Research Ethical Committee of China-Japan Union Hospital of Jilin University for Human Biomedical Research (2021080201) and informed consent was obtained from the participant. In accordance with the guidelines given by the ethical review board of China-Japan Union Hospital of Jilin University, a primary tumor sample was acquired from a pancreatic carcinoma volunteer (female), and PBMC was acquired from a healthy volunteer with HLA-A2 phenotype (female).

### Materials

DSPE-PEG_2000_ was purchased from Avanti Polar Lipids, Inc. Poly-L-lysine was purchased from Sigma-Aldrich. Agarose was purchased from Biowest. N2200 was purchased from Derthon Optoelectronics Materials Science Technology Company (Shenzhen, China). Tetrahydrofuran (THF) was purchased from J&K. RIPA Lysis Buffer, protein microBCA assay kit, and LIVE/DEAD™ cell imaging kit were purchased from Thermo Fisher (Massachusetts, USA). CCK-8 assay, 4’,6-diamidino-2-phenylindole (DAPI), Rhodamine-phalloidin, phosphate-buffered saline (PBS, pH = 7.4), and Phenylmethanesulfonyl fluoride (PMSF) were purchased from Solarbio Life Sciences (Beijing, China). Penicillin-streptomycin, RPMI 1640 medium, Dulbecco’s modified Eagle medium (DMEM) were purchased from Gibico (Gaithersburg, USA). Certified Fetal Bovine Serum (FBS) was purchased from VivaCell (Shanghai, China). LabPAGE 4–20% was purchased from Beijing LABLEAD BIOTECH Co., Ltd. (Beijing, China). The terminal deoxynucleotidyl transferase-mediated dUTP nick end-labeling (TUNEL) cell apoptosis detection kit was purchased from Beyotime Institute of Biotechnology (Shanghai, China). The polyfluorene derivative dye P-F8-DPSB (Ex: 405 nm, Em: 525 nm) was gifted from Prof. Fei Huang in South China University of Technology^38^. HSPs inhibitors (Tanespimycin and KNK437) were purchased from MedChemExpress.

### Cell lines and primary cells

4T1 murine breast cancer cells (catalog no. CL-0007), CT26 murine colorectal cancer cell line (catalog no. CL-0071), and LLC murine lung cancer cells (catalog no. CL-0140) were purchased from Procell Life Science&Technology Co., Ltd (Wuhan, China). Luc-4T1 cells (catalog no. LZQ0016) and Luc-Pan02 murine pancreatic adenocarcinoma cells (catalog no. LZQ0041) were purchased from Zhong Qiao Xin Zhou Biotechnology Co., Ltd (Shanghai, China). Furthermore, 4T1 cells, Luc-4T1 cells, CT26 cells were cultured and maintained in RPMI 1640 culture medium, while LLC cells and Luc-Pan02 cells were cultured and maintained in DMEM. Both media were supplemented with 10% FBS and 1% penicillin-streptomycin. All cells were cultured in an atmosphere at 37 °C with 5% CO_2_.

### Mice

BALB/c mice (6–8 weeks, female) and C57BL/6 (6–8 weeks, male) mice were obtained from Vital River Laboratories. Ifng-IRES-Venus-AkaLuci mice were obtained from Shanghai Model Organisms Center. NOD.Cg-Prkdc^scid^Il2rg^tm1Sug^/ShiJic (NTG) mice (6–8 weeks, female) were purchased from SiPeiFu Biotechnology. CD4-KO and CD8-KO mice sourced from the Jackson Laboratory. All the model mice were raised in a standard environmentally controlled room (23 °C, with 55 ± 5% humidity and under a 12–12 h light-dark cycle). when the mice received NIR laser irradiation, the distance between light source and mice was uniformed at 8 cm by a ruler, and the temperature of vaccination site was monitored by infrared infrared thermal camera over time. According to the animal ethics of our institute and animal welfare, animals were euthanized when their tumor volumes exceeded 1200 mm^3^ in all experiments of tumor inhibition.

### Preparation and characterizations of NPs

To synthesize photothermal nanoparticles (NPs), 400 μL of THF solution containing 2 mg DSPE-PEG_2000_ and 1 mg photothermal polymer N2200 was rapidly injected into 10 mL ultrapure water containing 1.25 mg PLys under ultrasound. Then, THF was blown off under nitrogen condition. The photothermal nanoparticles with negative charge (N-NPs) were prepared using the same method without adding PLys by using the same method. To synthesize fluorescent-labeled NPs, 0.1 mg fluorescent polymer P-F8-DPSB was supplemented in 400 μL of THF solution. Then, the morphologies of NPs and N-NPs were observed by transmission electron microscope (TEM) using HT7000 microscope (Hitachi, Japan) with 80 kV. Dynamic light scattering (DLS, Malvern ZEN 3600 Zetasizer, UK) with associated software (NANO ZS, version 7.12) was used to measure the hydrodynamic diameter and zeta potential of NPs and N-NPs.

### Preparation of fluorescent-labeled NPs

To synthesize fluorescent-labeled photothermal nanoparticles (NPs), 400 μL of THF solution containing 2 mg DSPE-PEG2000, 1 mg photothermal polymer N2200 and 0.1 mg fluorescent polymer P-F8-DPSB was rapidly injected into 10 mL ultrapure water containing 1.25 mg PLys under ultrasound. Then, THF was blown off under nitrogen condition.

### Evaluation of near-infrared performance of NPs

To evaluate photothermal performance, a series of nanoparticle aqueous solutions with different concentrations were continuously irradiated (808 nm, 0.65 W/cm^2^) for 1000 s. The temperatures were recorded by an infrared thermal camera (FLIR E40, USA) and treated with associated software (FLIR Tools, version 4.0.13330.1003) every 15 s during NIR laser irradiation. Furthermore, through treating the aqueous solutions of NPs (10 μg/mL) with 4 heating-cooling cycles, the corresponding temperatures were recorded and further made to curve for investigating their photothermal stability. All the experiments for evaluating NIR photothermal performances were carried out at the same room temperature. For evaluating photoacoustic property of NPs, the aqueous solutions of NPs ranging from 0 to 100 μg/mL (0, 25, 50, 75, 100 μg/mL) were put into the pre-prepared agarose gel molds and scanned using a multispectral optoacoustic tomography imaging system (MOST in Vision 128, iThera, Germany) to acquire the photoacaustic (PA) signals and PA images at 808 nm.

### Evaluation of cellular uptake

To investigate the cellular uptake, 4T1 cells were seeded onto 35 mm glass-bottom dishes at a density of 1 × 10^5^ cells incubated with NPs and N-NPs (10 μg/mL) at 37 °C for 4 h, respectively. Next, the cells were fixed by 4% paraformaldehyde at 37 °C for 20 min and then cell membrane was stained with rhodamine-phalloidin (excitation: 561 nm, emission: 595 nm) at 37 °C for 30 min before imaging by confocal laser scanning microscope (CLSM, Nikon A1, Japan) with associated software (NIS-Elements AR 5.20.00). NPs were excited at 405 nm, and images were captured at 510–540 nm. Furthermore, the 4T1 cells were seeded onto 24-well plate and incubated with NPs and N-NPs (10 μg/mL) for 4 h, respectively. Subsequently, the amounts of NPs and N-NPs internalized in cells were determined using a flow cytometer (Cytoflex LX, Beckman, USA) with associated software (version 2.3.1.22). The NPs with positive charge almost all internalized by tumor cells.

### Evaluation of photothermal effect of NPs in cells

4T1 cells were seeded onto 96-well plate at a density of 1 × 10^4^ cells and were treated with four different treatments: (1) PBS, without NIR laser irradiation; (2) PBS, NIR laser irradiation; (3) 10 μg/mL NPs-containing RPMI 1640 culture medium, without NIR laser irradiation; (4) 10 μg/mL NPs-containing RPMI 1640 culture medium, NIR laser irradiation. Before irradiation, the cells had been incubated with NPs for 12 h, and the free NPs had been washed off by PBS. Irradiation was provided by 808 nm laser with 0.65 W/cm^2^ for 40 min. Then, the temperature of with indicated treatments was recorded by the infrared thermal camera.

### Evaluation of photothermal effect on cell viability

For comparing the viabilities of tumor cells before and after NIR laser irradiation, 4T1 cells were seeded as described above and were treated with NPs or NPs+L (808 nm, 0.65 W/cm^2^, 40 min). Then, fresh medium containing the CCK-8 solution was added and cells were incubated for another 2 h. Finally, cell viability was measured using an Infinite M200 microplate spectrophotometer.

### Evaluation of the HSPs expression

4T1 cells were treated with indicated treatment as above section. Later, cells were washed with cold PBS, lysed with RIPA lysis buffer containing 1% PMSF on ice for 5 min, and centrifuged at 14,000 × *g* for 10 min. Supernatants were collected and the protein content was measured using a protein microBCA assay kit (Invitrogen). Supernatants from different groups were diluted to same protein concentration and mixed with SDS loading buffer before heating at 95 °C for 5 min. Samples were added (20 μL per well) and separated on LabPAGE 4–20% 15 Wells gel electrophoresis gels before being transferred to Immobilon-P polyvinylidene difluoride transfer membranes. After blocking in 5% nonfat dry milk for 2 h, membranes were incubated with primary antibody. Primary antibodies were diluted according to corresponding dilutions: GAPDH (1:3000, AF7021), HSP 70 (1:5000, ab194360), HSP 90 (1:5000, ab87133), HSP 105 (1:5000, ab109624). Then, membranes were incubated with HRP (Horseradish peroxidase) conjugated secondary antibody (1:2000, S0001) and imaged by using a multicolor fluorescent gel imaging system (DNR MF ChemiBIS3.2, version 7.0.12, Israel). The protein expression of LN-TCV-derived different cells (CT26, LLC, Luc-Pan02, and patient-derived tumor cells) also were evaluated by the same method and relatively quantified via calculating grayscale values by ImageJ software. For precise quantification, the concentrations of various HSPs in LN-TCV and PBS groups were executed by using ELISA assay. For verifying the impact of freeze-thaw process on HSPs upregulation, the expression of HSPs in LN-TCV receiving the freeze-thawing was also detected by western blotting and quantified via calculating grayscale values by ImageJ software. To further investigate whether non-tumor cells produced HSPs, myoblast cells (C2C12 cells), as the non-tumor cells, were treated with the temperature of the medium maintaining at 41 °C for 20 min and 40 min. Then, the expression levels of HSPs in cells and the concentration of HSPs in supernatant were detected by western blotting and ELISA, respectively.

### Evaluation of inactivation conditions

After treated with different formulations, 4T1 cells were treated with several freeze-thaw cycles (freeze at −20 °C for 2 h and thaw at 37 °C for 5 min) and the CCK-8 assay was used to evaluate the cell viability after different cycles. The freeze temperature (−20 °C) and two freeze-thaw cycles were chosen for maintaining the intact cell membrane after inactivation. The cells before freeze-thaw cycle and cultured 1, 2, 4 days after two freeze-thaw cycles were collected and stained by LIVE/DEAD™ cell imaging kit for further imaging by CLSM.

### Preparation and characterizations of LN-TCV

NPs-loaded-4T1 cells after NIR laser irradiation (808 nm, 0.65 W/cm^2^, 40 min) were digested and collected in PBS. And the LN-TC was activated via twice freeze-thaw cycles. To evaluate the cell membrane integrity of the LN-TCV, the fluorescence intensity of NPs in NPs-loaded cells and LN-TCV were quantitatively analyzed by FCM. To visualize the cell membrane framework of the LN-TCV, the cell membranes were stained by rhodamine-phalloidin before imaging. Moreover, FCM and CLSM were utilized for analyzing cell membrane integrity. The freeze-thaw treated cells were stained with propidium iodide (PI), and then these cells were detected by FCM for quantifying the positive signal. The nucleus in these cells also labeled by Hoechst 33342 for CLSM imaging. In the cell experiments, NPs were labeled by doping with an aggregation-induced emission dye (405 nm/525 nm).

To evaluate the photothermal of LN-TCV, 1 mL PBS solution containing LN-TCV (1 × 10^7^ cells/mL) was irradiated by continuous NIR laser irradiation (808 nm, 0.65 W/cm^2^) for 15 min. The infrared thermographic images and temperatures were obtained using infrared thermal camera. Moreover, PA images of LN-TCV with different concentrations (0, 2.5 × 10^6^, 5 × 10^6^, 7.5 × 10^6^, 1 × 10^7^ cells/mL) were obtained by using MOST. Moreover, CT26 cell-derived LN-TCV, LLC cell-derived LN-TCV, Luc-Pan02 cell-derived LN-TCV, and patient tumor cell-derived LN-TCV were prepared and characterized by the same method.

### Evaluation of photothermal effect at vaccination site

To test the photothermal effect in vivo, BALB/c mice were divided into four groups: (1) PBS (100 μL PBS), (2) LN-TCV (100 μL LN-TCV), (3) 1^st^ NIR irradiation (100 μL LN-TCV, NIR laser irradiation, 20 min, day 0), (4) 2^nd^ NIR laser irradiation (100 μL LN-TCV, twice NIR laser irradiation, 20 min, day 0 and day 5). LN-TCV (1 × 10^7^ cells/mL) was dispersed in PBS solution and administrated by subcutaneous injection at the back of mice. Then, irradiations were provided by 808 nm laser (0.65 W/cm^2^) to the same site of administration, and the infrared thermographic images of different groups were obtained using an infrared thermal camera. Specially, the infrared thermographic images of 2^nd^ NIR irradiation group were captured during second irradiation at day 5.

### Evaluation of long-time retention time of LN-TCV vaccine at vaccination site

BALB/c mice were injected subcutaneously with 100 μL of Cy5.5- succinimidyl ester (SE) labeled LN-TCV and divided into two groups: (1) LN-TCV, (2) LN-TCV + L. The mice in the group of LN-TCV + L were irradiated by 808 nm laser (20 min, 0.65 W/cm^2^). The retention time of NPs at the back of mice was also investigated upon the same equal dosage (10 μg/mL NPs), injection/NIR site (the back of mice), and NIR conditions (0.65 W/cm^2^, 20 min). In vivo imaging system (FX Pro, Kodak, USA, version 5.4.2.18893) was utilized to obtain the fluorescence images in both groups over time.

### Evaluation of clearance pathway of NPs

For evaluating the clearance pathway of NPs, the amount of N2200 in the feces was detected by using high-performance liquid chromatography (HPLC) assay. Specifically, the feces were collected from the mice receiving LN-TCV vaccination every week and further N2200 polymer in feces was extracted by dichloromethane. Then, the dried samples were re-suspended with acetonitrile solution before HPLC testing. In detail, the mobile phase was composed of 95% acetonitrile and 5% water. The column was eluted at a flow rate of 1 mL/min at 40 °C.

### Visualization of locally inflammation environment using transgenic mice

The Ifng-IRES-Venus-AkaLuci mice (6–8 weeks, female) were firefly Ifng-IRES-Venus-AkaLuciferase gene expression in IFN-γ reporter. The biodistribution and expression level of IFN-γ could be observed by the bioluminescence. The mice were divided into four groups as follows: (1) PBS, (2) LN-TCV, (3) LN-TCV + L, and (4) LN-TCV + 2L. The mice in PBS group were injected with 100 μL PBS, while that in other groups were injected with 100 μL LN-TCV (1 × 10^7^ cells/mL) at the back of mice. Next, the vaccination site of mice in LN-TCV + L, and LN-TCV + 2L were irradiated with 808 nm laser (0.65 W/cm^2^, 20 min) at day 0, and that of LN-TCV + 2L had another irradiation at day 5. The mice were injected with Aka Lumine n-Hydrochloride (i. p.) and visualized by the IVIS 200 imaging system (PerkinElmer, USA, version 4.5.5) every day.

### RNA-seq analysis

The BALB/c mice (4–6 weeks, female) were randomly divided into the same four groups and receiving corresponding treatments as above. Total RNA of different treatments was extracted using Trizol reagent kit (Invitrogen, Carlsbad, CA, USA) according to the manufacturer’s protocol. RNA quality was assessed on an Agilent 2100 Bioanalyzer (Agilent Technologies, Palo Alto, CA, USA) and checked using RNase-free agarose gel electrophoresis. After total RNA was extracted, eukaryotic mRNA was enriched by Oligo (dT) beads. Then the enriched mRNA was fragmented into short fragments using fragmentation buffer and reversely transcribed into cDNA by using NEB Next Ultra RNA Library Prep Kit for Illumina (NEB #7530, New England Biolabs, Ipswich, MA, USA). The purified double-stranded cDNA fragments were end repaired, A base added, and ligated to Illumina sequencing adapters. The ligation reaction was purified with the AMPure XP Beads (1.0X). And polymerase chain reaction (PCR) amplified. The transcriptome sequencing was performed using Illumina HiSeq TM2500 (Gene Denovo Biotechnology Co. China). RNA expression levels were determined using the fragments per kilobase of transcript per million mapped reads method. The fold-change method was used to identify RNAs that were differentially expressed after castration using the R package DEGseq (R-3.6.2). Genes with a log2 fold change >2 and an adjusted *p* < 0.05 were deemed to be significantly differentially expressed. The typical data from the top 620 gene clusters were presented. The typical data from top 40 GSEA were presented.

### Evaluation of recruitment and maturation of DCs at vaccination site

BALB/c mice (4–6 weeks, female) were also divided into the same groups (PBS, LN-TCV, LN-TCV + L, and LN-TCV + 2L) and receiving corresponding treatments as above. Then, mice in different groups were sacrificed at the indicated time points (day 1, 2, 3, 4, 5, 6, 7, 8, 9, and 10), and the tissues at vaccination sites were collected, ground up, sieved, and stained with PE-conjugated anti-mouse CD11c were analyzed by FCM. To evaluate DCs recruitment, the vaccination sites in each group were harvested at day 6, frozen in OCT (optimal cutting temperature) and sectioned into 10-μm-thick slices. After fixing in 4% polyoxymethylene and blocking with BSA (3%), the sections were incubated with mouse monoclonal to CD11c (1:100, ab254183) and then incubated with corresponding secondary antibody (1:2000, A-11001). After that, the sections were stained with DAPI, and then analyzed by automatic multispectral imaging system (PerkinElmer Vectra II, USA). To further assess DCs recruitment and activation, the tissues from vaccination sites were collected, ground up, sieved, and stained with Ghost dye (UV405). The single-cell suspension was incubated with anti-CD16/32 and then stained with FITC-conjugated anti-mouse CD11c, BV605-conjugated anti-mouse MHC I, PE-Cy7-conjugated anti-mouse CD80, APC-conjugated anti-mouse CD40, and PerCP-Cy5.5-conjugated anti-mouse CD86 antibodies. Then, the stained cells measured by FCM.

### PA imaging of LN-TCV in lymph node

BALB/c mice (4–6 weeks, female) were randomly divided into indicated groups (PBS, LN-TCV, LN-TCV + L, and LN-TCV + 2L) and received corresponding treatments as above. For PA imaging, the mice were anaesthetized and imaged the inguinal lymph node via (LAZR-X Vevo, FUJIFILM VisualSonics, Canada) at day 9.

### Evaluation of immune responses in inguinal lymph node

BALB/c mice (4–6 weeks, female) were randomly divided into indicated groups (PBS, LN-TCV, LN-TCV + L, and LN-TCV + 2L) and received corresponding treatments as above. Next, the inguinal lymph nodes were collected from each mouse at day 9. The single-cell suspensions were also obtained using the method described above. To assess the maturation of DCs, the cells firstly stained with Ghost Dyes at 4 °C for 30 min. The single-cell suspension was incubated with anti-CD16/32 and then the cells were further stained with FITC-conjugated anti-mouse CD11c, PE-Cy7-conjugated anti-mouse CD80, and PerCP-Cy5.5-conjugated anti-mouse CD86 antibodies at 4 °C for 30 min. To examine the proliferation of T cells, the cells firstly stained with Ghost Dyes at 4 °C for 30 min. The single-cell suspension was incubated with anti-CD16/32 and then cells were further stained with PE-conjugated anti-mouse CD3, BV605-conjugated anti-mouse CD8. After that, these cells were punched and then stained with PerCP-cy5.5-conjugated anti-mouse IFN-γ and Alexa Flour647-conjugated anti-mouse granzyme B. All samples were measured by FCM. For microscopically observing the proliferated cells and the increase of CD8 + T cells, lymph node sections by immunostaining of Ki67 and CD8 were executed. The sections were stained with antibody to CD8 (1:50, 14-0081-82) and Ki67 (1:100, ab16667) and then incubated with secondary antibody (1:300, ab150154 and 1:300, S0008) before CLSM observation.

### Evaluation of the immunogenicity of HSPs

Prior to investigate the release of HSPs, HSP 70 with the most well-known adjuvanticity was engineered and fused with mCherry in 4T1 tumor cells. All DNA sequences used to engineer the 4T1 cells with the expression of HSP-mCherry were provided in Supplementary Table 3. Then, the engineered LN-TCV was vaccinated at the back of mice and the signal of mCherry in the DCs in the skin was detected by imunohistochemical analysis. In order to exclude the link between other mechanisms and immunogenicity, high mobility group protein 1 (HMGB1) and calreticulin (CRT) in the LN-TCV group and overheating group were investigated. Tumor cells were seeded as described above and treated with various conditions (41 °C, 40 min for LN-TC; 45 °C, 40 min as positive control). Then these cells were stained with anti-mouse HMGB1 and anti-mouse CRT at 37 °C before CLSM observation, respectively. Besides, the inhibitors (Tanespimycin and KNK437) were utilized for blocking the HSPs expression in constructing LN-TCV. And this vaccine was incubated with DCs and the activation and presentation were investigated by FCM.

### CDR3 high-throughput sequencing

BALB/c mice (4–6 weeks, female) were also divided into the same groups (PBS, LN-TCV, LN-TCV + L, and LN-TCV + 2L) and received corresponding treatments as above. RNA in lymph nodes from indicated groups was extracted by using RNeasy Plus mini kit (Qiagen). Then, samples were analyzed by high-throughput sequencing of TCR using the ImmuHub^TM^ TCR profiling system (ImmuQuad Biotech, Hangzhou, China). Briefly, the sequencing was performed on an Illumina HiSeq X10 system with PE150 mode (Illumina) by using a 5′ RACE unbiased amplification protocol. A post sequencing algorithm was applied to raw sequencing data for PCR and sequencing errors correction and V, D, J, C gene segments mapping with IMGT. The resulting nucleotide and amino acid sequences of CDR3 of TCRβ were determined, and those with out-of-frame and stop codon sequences were removed from the identified TCRβ repertoire. We further defined the amounts of each TCRβ clonotype by adding numbers of TCRβ clones sharing the same nucleotide sequence of CDR3.

### Antitumor study in 4T1 breast tumor model

BALB/c mice (4–6 weeks, female) were subcutaneously inoculated with 1 × 10^6^ 4T1 cells at the ventral mammary fat pad on day −7. When the tumor volume developed to around 60 mm^3^ at day 0, mice were separated randomly into five groups (*n* = 6) and treated as follows: (1) PBS, (2) LN-TCV, (3) LN-TCV + L, (4) LN-TCV + 2L, (5) TCV + 2L, and (6) N-TCV + 2L. The mice in PBS group were subcutaneously injected with PBS, whereas that in other groups were vaccinated with LN-TCV or TCV at the back of tumor-bearing mice. For NIR treatment, the mice in LN-TCV + L, LN-TCV + 2L, and TCV + 2L groups were anaesthetized and irradiated with 808 nm laser (0.65 W/cm^2^, 20 min) at the vaccination sites for 20 min at day 0, and the mice in LN-TCV + 2L, and TCV + 2L groups had another irradiation at day 5. Furthermore, TCV + BCG (10^7^ TCV and 10^8^ organism BCG per 1 mL) was used as a standard treatment, in which tumor-bearing mice were vaccinated at day 0 and day 5. Then, N-TCV + BCG + 2L (10^7^ cells N-TCV and 10^8^ organism BCG per 1 mL) also was used as a control, in which tumor-bearing mice were vaccinated at day 0 and irradiated with NIR laser at day 0 and day 5. Tumor size was measured every 2 days by digital caliper and the tumor volume (mm^3^) was calculated by using the formula: volume (mm^3^) = width^2^ × length × 0.5. Then, the tumor tissues in different treatments were obtained for imunohistochemical analysis via the method described above. Proliferating cell nuclear antigen-Ki67 was used for determining nucleus proliferating of tumor tissue. What’s more, the tumor metastasis sites in lung, appearing as yellow nodules, were counted and the sections of lung tissue were stained with H&E and imaged via Vectra II. And the tumor metastasis in tibias were scanned by Computed tomography (CT).

### Antitumor study in Luc-4T1 recurrent tumor model

To mimic the residual microtumor in the surgical bed in clinic, BALB/c mice (4–6 weeks, female) were subcutaneously inoculated with 1 × 10^6^ Luc-4T1 cells at the ventral mammary fat pad on day −10 and most tumor was resected when the volume of tumor arrive at about 200 mm^3^. The postoperative mice whose tumors were extremely small or large were excluded from enrollment by the mean of bioluminescence imaging. The mice were randomly divided into three groups and treated as follows: (1) PBS, (2) LN-TCV + L, (3) LN-TCV + 2L. For NIR treatment, the mice in LN-TCV + L and LN-TCV + 2L were anaesthetized and irradiated with 808 nm laser (0.65 W/cm^2^, 20 min) at day 0, and the mice in LN-TCV + 2L had another irradiation at day 5. The tumor size was recorded and the tumor growth was monitored through the bioluminescence images by using IVIS 200 imaging system at day −3, day −2, day 10, and day 20 after intraperitoneally injected with luciferin and anaesthetized by isoflurane. Besides, a part of the mice in different groups was sacrificed and spleens were collected, ground up, sieved, and stained for analyzing memory T cells by FCM.

### On-demand antitumor study in diverse tumor model

Mice were subcutaneously inoculated at the abdomen of mice with CT26 cells (1 × 10^6^ cells per mouse, BALB/c, 4–6 weeks, female), LLC cells (1 × 10^6^ cells per mouse, C57BL/6, 4–6 weeks, male), and Luc-Pan02 cells (1 × 10^6^ cells per mouse, C57BL/6, 4–6 weeks, male), respectively. Three types tumor-bearing mice were treated with corresponding tumor cell-derived LN-TCV and irradiated by NIR laser according to an on-demand irradiation regime. For giving NIR laser irradiations with a rationally appropriate times and intervals, FTGR (fluctuation of tumor growth rate) was proposed to monitor the tumor growth and calculated by the equation:

$${{{{{\rm{FTGR}}}}}}=\{[{{{{{\rm{V}}}}}}({{{{{{\rm{t}}}}}}}_{{{{{{\rm{n}}}}}}+2})-{{{{{\rm{V}}}}}}({{{{{{\rm{t}}}}}}}_{{{{{{\rm{n}}}}}}})]/{{{{{\rm{V}}}}}}({{{{{{\rm{t}}}}}}}_{{{{{{\rm{n}}}}}}})*100\%\}-\{[{{{{{\rm{V}}}}}}({{{{{{\rm{t}}}}}}}_{{{{{{\rm{n}}}}}}})-{{{{{\rm{V}}}}}}({{{{{{\rm{t}}}}}}}_{{{{{{\rm{n}}}}}}-2})]/{{{{{\rm{V}}}}}}({{{{{{\rm{t}}}}}}}_{{{{{{\rm{n}}}}}}-2})*100\%\}$$

V(t_n_) = mean tumor volume at time t_n_.

According to the value of FTGR, the mice in LN-TCV + 2L or LN-TCV + 3L group were given another irradiation (20 min, 0.65 W/cm^2^) when the value excessed 0. In the experiment, six tumor-bearing mice (tumor volume reached about 60 mm^3^) were randomly divided into PBS group and the rest mice received LN-TCV (100 μL, 1 × 10^7^ cells/mL LN-TCV) vaccination and irradiation at day 0. After treatment, tumor sizes were serially measured with a digital caliper every 2 days. Then, the value of FTGR was calculated by the equation to estimate on-demand irradiation times and intervals:

1. when the value of FTGR excessed 0, six mice received LN-TCV vaccination and the first irradiation was divided into LN-TCV + L group and the rest mice received another irradiation (20 min, 0.65 W/cm^2^).
2. when the value of excessed 0 again, six mice received LN-TCV vaccination and the second irradiation was divided into LN-TCV + 2L group and the rest mice received another irradiation (20 min, 0.65 W/cm^2^).

For the CT26 and LLC tumor model, twice NIR laser irradiation was enough to keep the values of FTGR below 0. Furthermore, we also executed at impropriate time points in CT26 tumor model to evaluate the rationality of utilizing FTGR value for executing on-demand NIR laser irradiation. Whereas, mice in Luc-Pan02 tumor model needed to execute three times NIR laser irradiation to keep the value of FTGR below 0. According to FTGR value, the time point of second or third NIR laser irradiation was late in the on-demand antitumor study in diverse tumor model. Thus, considering the metabolism of LN-TCV over time, the temperature of vaccination site was monitored by infrared thermal camera and maintained around 41 °C by tuning the power of NIR laser irradiation.

Meanwhile, the tumor growth of Luc-Pan02 tumor model was monitored through the bioluminescence images at day 0, day 7, day 14, and day 21. To evaluate the tumor cell apoptosis, TUNEL analysis was used to stain the section of tumor tissues from different groups in all three tumor models. To evaluate the tumor microenvironment amelioration effect of LN-TCV, tumor sections were stained by various antibodies. All samples were measured by FCM.

### Antitumor study in tumor-bearing (CD8-KO and CD4-KO) mice

Luc-Pan02 tumor cells were inoculated in C57BL/6 (4–6 weeks, male), CD8-KO (4–6 weeks, male), and CD4-KO (4–6 weeks, male) mice, respectively. Tumor-bearing C57BL/6 mice were divided into PBS (100 μL, PBS) and LN-TCV + 3L (100 μL, 1 × 10^7^ cells/mL LN-TCV + 3 NIR laser irradiation) groups. Then, both tumor-bearing CD8-KO and CD4-KO mice were vaccinated with LN-TCV and three times NIR laser irradiation at indicated time. Tumor size and survival time were recorded and the tumor growth was monitored through the bioluminescence images at day 0, day 7, day 14, and day 21.

### Evaluation of specificity antitumor effect of LN-TCV

C57BL/6 mice (4–6 weeks, male) were incubated with Luc-Pan02 tumor cells and divided into three groups and treated as follows: (1) PBS (100 μL, PBS); (2) LN-TCV(4T1) + 3L (100 μL, 1 × 10^7^ cell/mL LN-TCV + three times NIR laser irradiation); (3) LN-TCV(Luc-Pan02) + 3L (100 μL, 1 × 10^7^ cell/mL LN-TCV + three times NIR laser irradiation). Then, tumor size and survival time were recorded.

### Antitumor study in humanized patient-derived tumor xenograft (PDX) model

For humanized PDX construction, a primary tumor sample was resected from a patient with pancreatic cancer and engrafted to mice for three passages. Then, the tumor sample subcutaneously transplanted into the axilla of the NOD-Prkd^em26^Il2rg^em26^/Nju (NTG) (4–6 weeks, female), and peripheral blood mononuclear cells (PBMCs) (1 × 10^6^ cells per mice), which were extracted from the human peripheral blood of healthy donor, were intravenously injected into tumor-bearing NTG mice. Peripheral blood from all mice was monitored for lymphocyte (hCD45^+^) reconstitution at the indicated time. After four weeks, the tumor tumors were visible (~60 mm^3^) and the proportion of human lymphocyte in mice peripheral blood arrived to 25%, representing the success of humanized PDX mice construction. Before treatment for humanized PDX mice, the patient-derived LN-TCV (LN-pTCV) was constructed. In detail, the small tumor sample was digested by collagenase (type I) at 37 °C for 30 min. Then the mixed cells were centrifuged and the supernatant was discarded. Then the patient-derived tumor cells were obtained and seeded in 6-well plates. After that, the similar construction method of LN-TCV was proceeded in patient-derived tumor cells. Later, the mice were treated with LN-pTCV and on-demand irradiation regime according to FTGR. At day 16, the sections of tumor tissues from different groups were prepared and stained for further analyzation.

### The evaluation for the safety of different tumor treatments

The main organs (heart, liver, spleen, and kidney) were sliced and stained by hematoxylin-eosin (H&E) staining. Besides, the serum levels of urea nitrogen (BUN), lactate dehydrogenase (LDH), alanine aminotransferase (ALT), aspartate transaminase (AST), and alkaline phosphatase (ALP) were analyzed by using an automated analyzer (Hitachi Ltd Hitachi-917, Japan).

### TGI calculation

TGI was calculated by the formula: TGI = [1−(T_t_/T_0_)/(C_t_/C_0_)]*100%, where T_t_ = tumor volume of treated group at time t, T_0_ = mean tumor volume of treated group after randomly grouping, C_t_ = tumor volume of PBS group at time t, C_0_ = mean tumor volume of PBS group after randomly grouping.

### Statistical analysis

Statistical analysis was performed using GraphPad Prism 9.0.0. Statistical analysis was performed using two-tailed Student’s t test (comparison between two groups) or one-way analysis of variance (ANOVA; comparison among more than two groups) or log-rank Mantel-Cox test (survival time).

### Reporting summary

Further information on research design is available in the Nature Portfolio Reporting Summary linked to this article.

## Supplementary information

Supplementary Information

Reporting Summary

## Data Availability

RNA-seq and CDR3 high-throughput sequencing datasets are available in NCBI under accession codes SRP390019 for RNA-seq and SRP449756 for CDR3 high-throughput sequencing. Source data for main and supplementary figures are provided with this paper and are also available in Figshare (10.6084/m9.figshare.23674254.v1). The remaining data are available within the Article, Supplementary Information or Source data file. Source data are provided with this paper.

## Source data

Source Data
