# Supplementary material for: Generation of whole tumor cell vaccine for on-demand manipulation of immune responses against cancer under near-infrared laser irradiation
Source: Nat Commun. 2023 Jul 26;14:4505. doi: 10.1038/s41467-023-40207-y (PMC10372023; doi:10.1038/s41467-023-40207-y)
Supplement: Supplementary file 1 — Supplementary Information [file 41467_2023_40207_MOESM1_ESM.pdf]

Supplementary Information

Generation of whole tumor cell vaccine for on-demand manipulation of immune responses against cancer under near-infrared laser irradiation

Author list: Jiaqi Meng[1,2#], Yanlin Lv[1#], Weier Bao[1,2#], Zihui Meng[3], Shuang Wang[1], Yuanbin Wu[1], Shuping Li[1], Zhouguang Jiao[1], Zhiyuan Tian[2*], Guanghui Ma[1,4*], Wei Wei[1,4*]

Affiliations:

[1]State Key Laboratory of Biochemical Engineering, Institute of Process Engineering, Chinese Academy of Sciences, Beijing 100190, P. R. China.

[2]School of Chemical Sciences, University of Chinese Academy of Sciences, Beijing 100049, P. R. China.

[3]Department of Hepatobiliary-Pancreatic Surgery, China-Japan Union Hospital of Jilin University, Changchun 130033, PR China.

[4]School of Chemical Engineering, University of Chinese Academy of Sciences, Beijing 100049, P. R. China.

#These authors contributed equally.

*These authors jointly supervised this work.

*Email: weiwei@ipe.ac.cn, ghma@ipe.ac.cn, zytian@ucas.ac.cn.

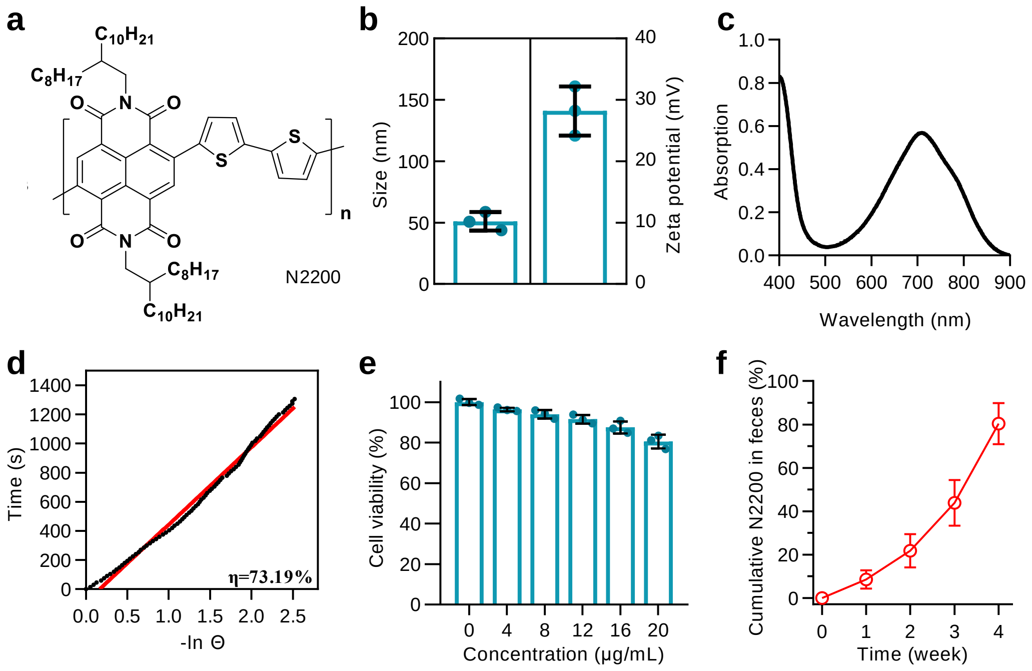

Supplementary Fig. 1. Supplementary data of characterizations of photothermal nanoparticles (NPs).

(a) The structural formula of photothermal polymer N2200 (poly[[1,2,3,6,7,8-hexahydro-2,7-bis(2-octyldodecyl)-1,3,6,8dioxobenzo [LMN] [3,8] phenanthroline-4,9-diyl] [2,2'-bithiophene]-5,5'-diyl]). The alternating donor-acceptor-donor system with thiophene rings as the donor and naphthalene diimide as the acceptor and the appropriate donor system size together intrinsically imparted relevant excitation with predominant charge transfer, which enabled efficient light-harvesting capacity.

(b) Size and zeta potential of NPs in aqueous solution. NPs exhibited positive charge about +28 mV and had an average hydrodynamic diameter of about 50 nm.

(c) The absorption spectrum of NPs (100 μg/mL) along the range of 400-900 nm. The absorption of NPs at 808 nm was benefited for subsequent converting the energy of NIR to heat.

(d) Calculation of photothermal conversion efficiency of NPs. The N2200-based NPs had an excellent photothermal conversion ability (η=73.19%).

(e) The cell viability of 4T1 cells incubated with NPs. NPs performed non-toxic to 4T1 tumor cells within 20 μg/mL after 24 h incubation, indicating that they had the good biocompatibility for further investigation.

(f) Cumulative quantification of N2200 polymer in feces after subcutaneous injecting LN-TCV. Most of N2200 polymer were detected in the feces, indicating the clearance pathway in vivo.

Data in b, e, and f were represented as mean values ± s.d., n = 3 biologically independent samples.

The experiments in c and d were repeated three times independently with similar results.

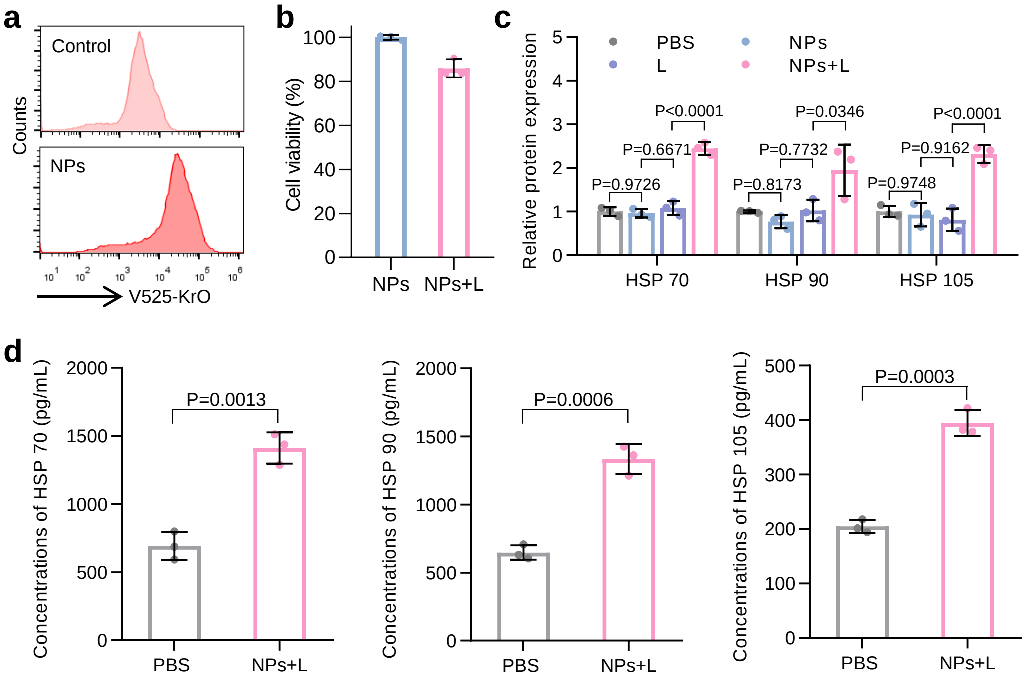

Supplementary Fig. 2. Supplementary data of the effect of the generation of heat shock proteins (HSPs) upon NIR laser irradiation.

(a) Flow cytometry analysis (FCM) for NPs internalized by 4T1 tumor cells. NPs were abundantly internalized by 4T1 tumor cells, benefiting for subsequent generating heat in cells upon low-power NIR irradiation.

(b) Cell viability of 4T1 tumor cells receiving various treatments (NPs and NPs+L). 40 min NIR laser irradiation had a very slight killing effect to the tumor cells.

(c) Relative HSPs expression in 4T1 tumor cells receiving different treatments. The upregulated the expression of HSPs showed the requirements for both NPs and NIR laser irradiation. The P values of NPs to PBS, L to NPs, and NPs+L to L were 0.9726, 0.6671, <0.0001 for the relative protein expression of HSP 70, 0.8173, 0.7732, 0.0346 for the relative protein expression of HSP 90, and 0.9748, 0.9162, <0.0001 for the relative protein expression of HSP 105, respectively.

(d) Enzyme linked immunosorbent assay (ELISA) of HSPs concentrations in PBS and NPs+L treatment. NIR laser irradiated N-TC strongly upregulated the expression of HSPs. The P values of NPs+L to PBS were 0.0013 for the concentrations of HSP 70, 0.0006 for the concentrations of HSP 90, and 0.0003 for concentrations of HSP 105, respectively.

Data in b, c, and d were represented as mean values ± s.d., n = 3 biologically independent samples.

P values in c were calculated by using one-way ANOVA.

P values in d were calculated by using two-tailed unpaired Student's t-test.

The experiment in a was repeated three times independently with similar results.

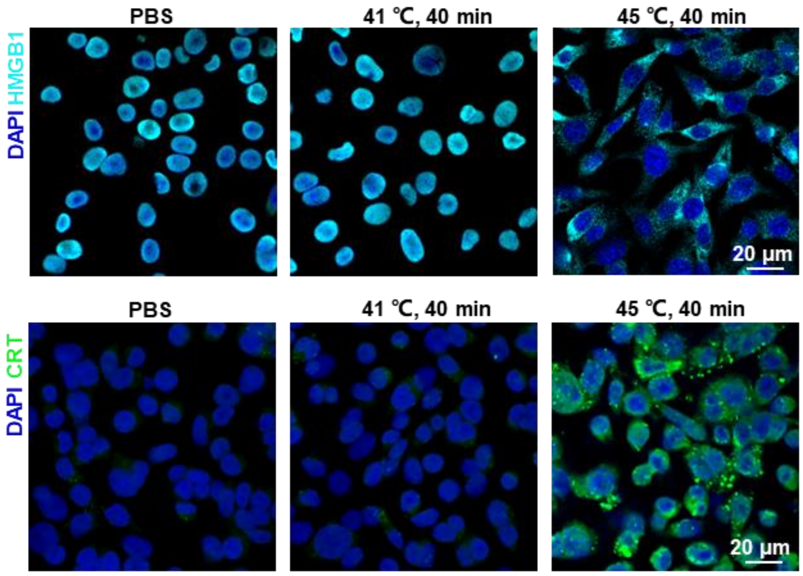

Supplementary Fig. 3. Supplementary data of confocal laser scanning microscopy (CLSM) images of in vitro high mobility group protein 1 (HMGB1) intracellular translocation or calreticulin (CRT) expression after different treatments (41 °C, 40 min for LN-TC; 45 °C, 40 min as positive control) in vitro. Neither intracellular translocation of HMGB1 nor upregulated expression of CRT was observed upon NIR laser irradiation, thus excluding the adjuvanticity of these two well-known damage-associated molecular patterns. The images were presented with same magnification. This experiment was repeated three times independently with similar results.

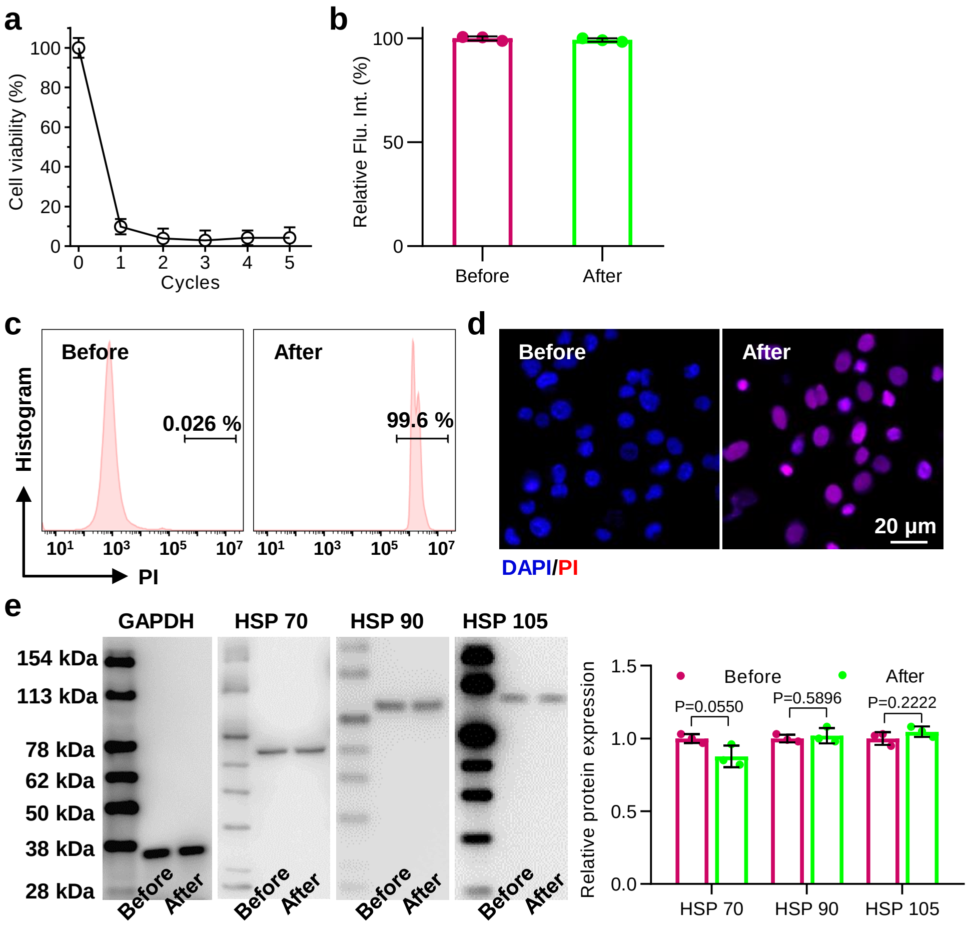

Supplementary Fig. 4. Supplementary data of the process of LN-TCV construction.

(a) The cell viability of LN-TCV with different freeze-thaw cycles. After two or more freeze-thaw cycles, almost no cells kept survival, indicating the safe use of LN-TCV with low tumorigenicity in vivo.

(b) The quantification of NPs leakage from cells before and after freeze-thaw process detected by FCM. After two freeze-thaw cycles, no NPs leaked out from cells, which indicated that the construction method of the LN-TCV could preserve the cell membrane framework.

(c) Propidium iodide signals in nucleus before and after freeze-thaw process detected by FCM.

(d) Propidium iodide signals in nucleus before and after freeze-thaw process detected by CLSM. The freeze-thaw process indeed opened the pores in the cell membrane, which could be indicated by the positive signal of propidium iodide in the nucleus. The images were presented with same magnification.

(e) **Western blotting analysis of the expression of HSP 70, HSP 90, and HSP 105 proteins in 4T1 tumor cells before and after freeze-thaw process.** The freeze-thaw process hardly induced upregulation of HSPs in LN-TCV. The P values of After to Before were 0.0550 for the relative protein expression of HSP 70, 0.5896 for the relative protein expression of HSP 90, and 0.2222 for the relative protein expression of HSP 105, respectively.

Data in a, b, and e were represented as mean values ± s.d., n = 3 biologically independent samples.

P values in e were calculated by using two-tailed unpaired Student's t-test.

The experiments in c, d, and e were repeated three times independently with similar results.

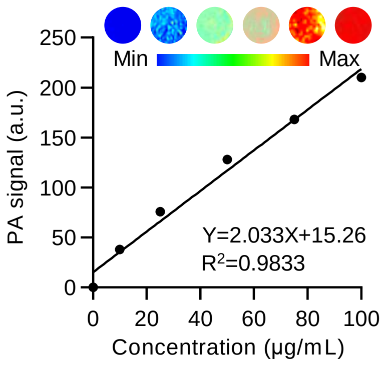

Supplementary Fig. 5. Supplementary data of in vitro representative photoacoustic (PA) images and the corresponding PA values of NPs at 808 nm. The PA signals of NPs were linearly related to the concentration, demonstrating the potential application of NPs for in vivo PA imaging.

The experiment was repeated three times independently with similar results.

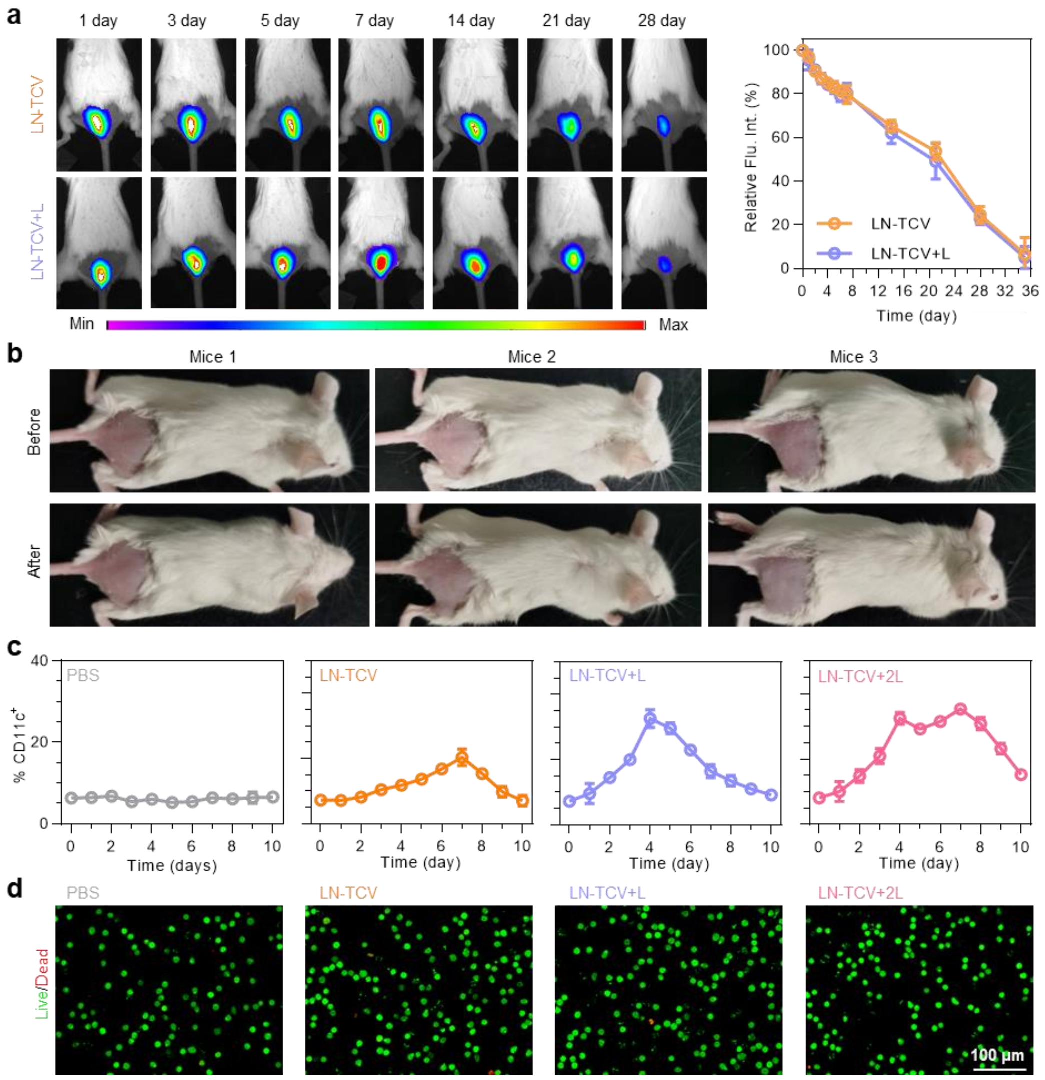

Supplementary Fig. 6. Evaluation of retention time of LN-TCV in vivo and recruitment of DCs at vaccination sites.

(a) In vivo animal fluorescence images (left) and quantitative analysis (right) at the vaccination sites after being injected with LN-TCV with or without NIR irradiation (808 nm, 0.65 W/cm^2, 20 min). LN-TCV was labelled by Cy5.5-SE. The LN-TCV could stay at the vaccination site for a long period with or without laser irradiation, benefiting for further NIR laser irradiation manipulating on-demand immune response for anti-tumor therapy. The images were representative of three independent mice.

(b) Photographs of mice before and after NIR laser irradiation (808 nm, 0.65 W/cm^2, 20 min) (n = 3 mice per group). The vaccination sites had no redness and swelling, thus indicating a good tolerance of mice to our mild NIR laser irradiation.

(c) **Quantitative FCM results for the dynamic of DCs recruitment to the vaccination sites in response to different treatments (PBS, LN-TCV, LN-TCV+L, and LN-TCV+2L) at indicated time.** After different vaccinations, all groups could induce different degrees of DCs recruitment. Specifically, the twice irradiation could recruit DCs again compared to mice received once irradiation at day 0.

(d) **Live/Dead analysis of subcutaneous cells in indicated groups.** After twice NIR laser irradiation, almost all recruited DCs kept alive, which was benefited for further antigen presentation and DCs homing. Green: live cells; Red: dead cells. The images were representative of three independent mice and were presented with same magnification.

Data in a and c were represented as mean values ± s.d., n = 3 mice per group.

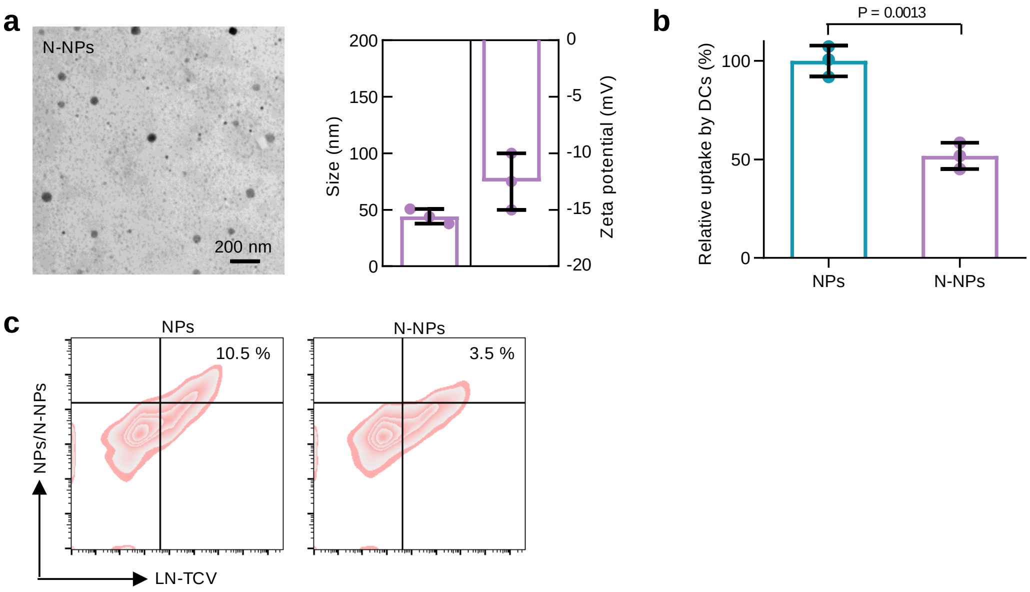

Supplementary Fig. 7. The advantages of positive charged NPs on BMDC uptake in vitro.

(a) Preparation of negative charge nanoparticles (N-NPs) as a counterpart. The N-NPs were uniformly dispersed in the field of view (left) and the N-NPs exhibited had an average hydrodynamic diameter of about 48 nm and charge of -12 mV (right).

(b) Uptake of NPs and N-NPs by bone marrow dendritic cells (BMDCs) by FCM. The P value of N-NPs to NPs was 0.0013.

(c) Representative FCM plot of the uptake of tumor cell fragments with NPs or N-NPs by BMDCs. Compared to the N-NPs, NPs were internalized by BMDCs with a larger amount, which further facilitated the uptake of tumor cell fragments by BMDCs.

Data in a and b were represented as mean values ± s.d., n = 3 biologically independent samples.

P values in b were calculated by two-tailed unpaired Student's t-test.

The experiments in a and c were repeated three times independently with similar results.

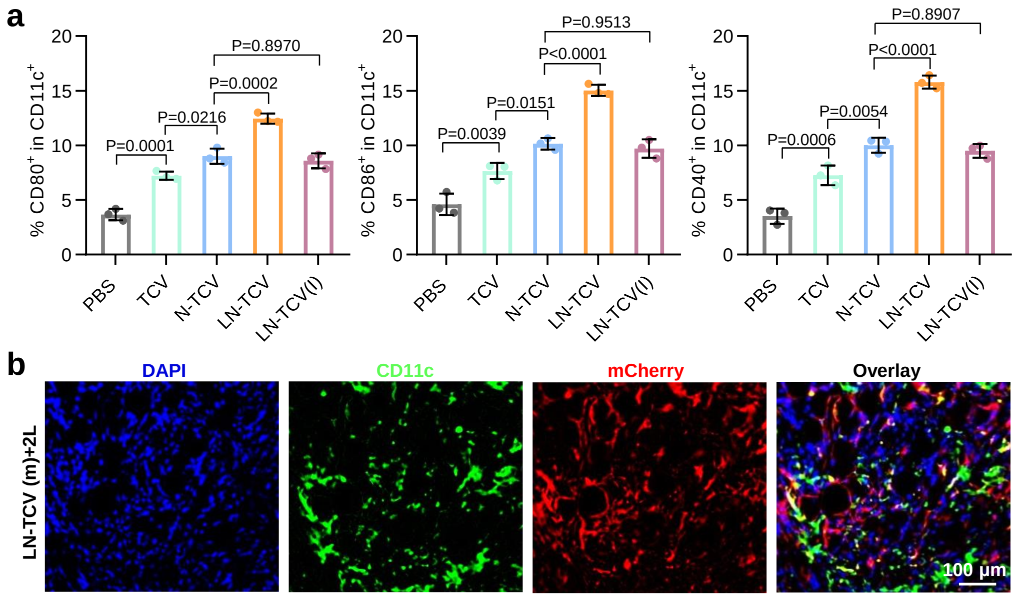

Supplementary Fig. 8. Evaluation of the link between HSPs expression and their immunogenicity.

(a) The flow cytometry analysis of DCs activation in vitro in various treatments. The HSPs inhibitors were incubated with the tumor cells before LN-TCV preparation in the LN-TCV(I) group. Compared with TCV and N-TCV (NPs-adopted TCV) groups, the DCs incubated with LN-TCV exhibited high levels of costimulatory molecules (CD40, CD80, and CD86), indicating that multiple endogenous HSPs producing by NIR laser irradiation promoted DCs activation and maturation. Once LN-TCV were pretreated with HSPs inhibitors, the positive percentages of costimulatory molecules on DCs were compromised. The P values of TCV to PBS, N-TCV to TCV, LN-TCV to N-TCV, and LN-TCV(I) to N-TCV were 0.0001, 0.0216, 0.0002, 0.8970 for the percentage of $CD80^+$ in $CD11c^+$, 0.0039, 0.0151, <0.0001, 0.9513 for the percentage of $CD86^+$ in $CD11c^+$, and 0.0006, 0.0054, <0.0001, 0.8907 for the percentage of $CD40^+$ in $CD11c^+$, respectively.

(b) Immunofluorescence slices of vaccination site. The signal of HSPs could be detected in DCs at the vaccination site, which could release and serve as endogenous adjuvants for DCs activation and maturation. Blue: nucleus; Green: DCs; Red: HSP-mCherry. The images were representative of three independent mice and were presented with same magnification.

Data in a were represented as mean values ± s.d., n = 3 biologically independent samples.

P values in a were calculated by using one-way ANOVA.

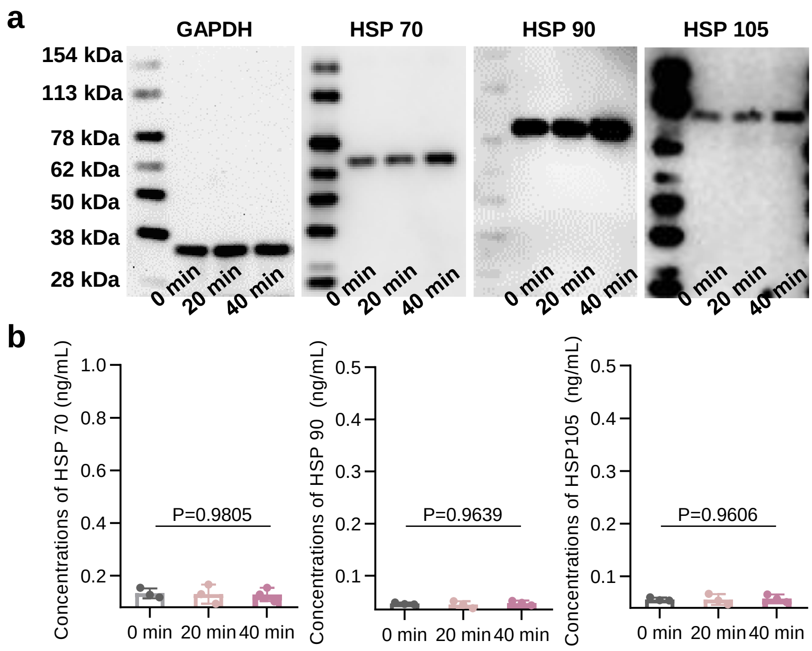

Supplementary Fig. 9. Investigation of the production and secretion of HSPs in non-tumor cells after mild heating.

(a) **Western blotting analysis of the expression of HSP 70, HSP 90, and HSP 105 proteins in C2C12 cells after different treatments.** No significant difference was observed in the expression of HSPs in the C2C12 cells between the groups without treatment and treated 41℃ for 20 min, while that in the C2C12 cells treated at 41℃ for 40 min showed significant upregulation. The result demonstrated that the NIR irradiation period at the vaccination site (20 min) was too short to promote the production of HSPs.

(b) **Concentrations of HSPs in the supernatant of C2C12 cells after different treatments.** The amounts of HSPs (HSP 70, HSP 90, and HSP 105) in the supernatant from all three groups were detected with very low levels and very slight variations, which excluded the possibility of releasing HSPs into the extracellular environment for DCs activation. The P values of 40 min to 0 min were 0.9807 for the concentrations of HSP 70, 0.9566 for the concentrations of HSP 90, and 0.9606 for concentrations of HSP 105, respectively.

Data in b were represented as mean values ± s.d., n = 3 biologically independent samples.

P values in b were calculated by using one-way ANOVA.

The experiment in a was repeated three times independently with similar results.

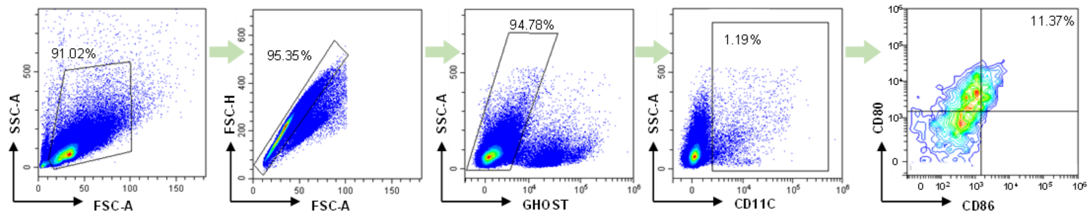

Supplementary Fig. 10. Flow cytometry gating strategies for mature DCs (CD80$^+$CD86$^+$ in CD11c$^+$ gate) in the lymph nodes (related to Fig. 3b).

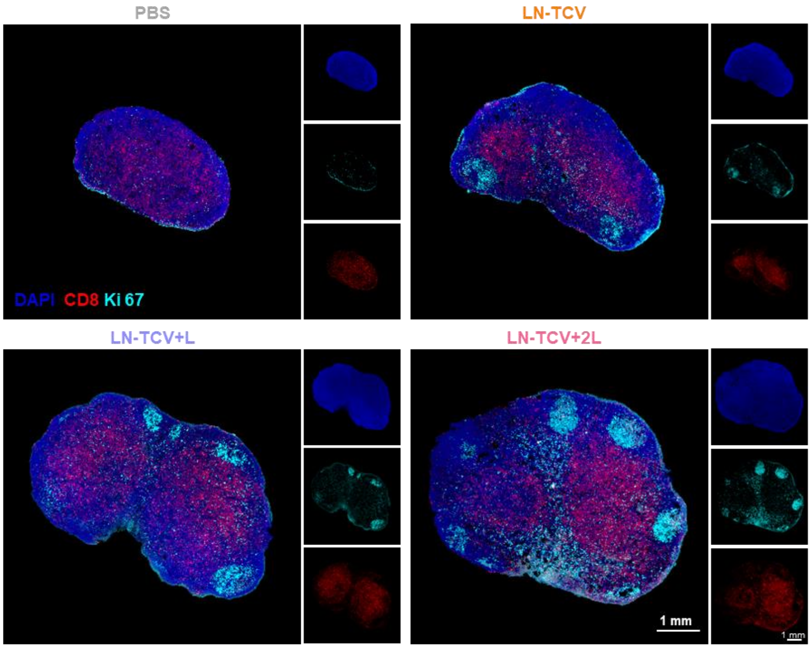

Supplementary Fig. 11. Immunofluorescence analysis of CD8^{+}T cells with distinct proliferation activity in the lymph nodes upon indicated treatments. Red: CD8^{+}T cells; Cyan: proliferated cells; Blue: cell nucleus. The images were representative of three independent mice and were presented with same magnification.

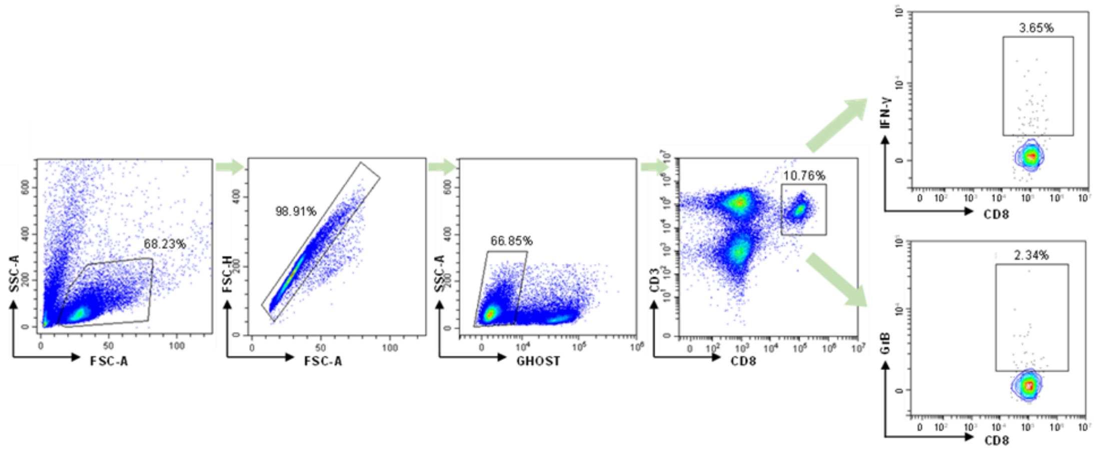

Supplementary Fig. 12. Flow cytometry gating strategy for evaluating IFN-γ-producing and granzyme B-producing in CD8⁺ T cells in the lymph nodes (related to Fig. 3d and 3e).

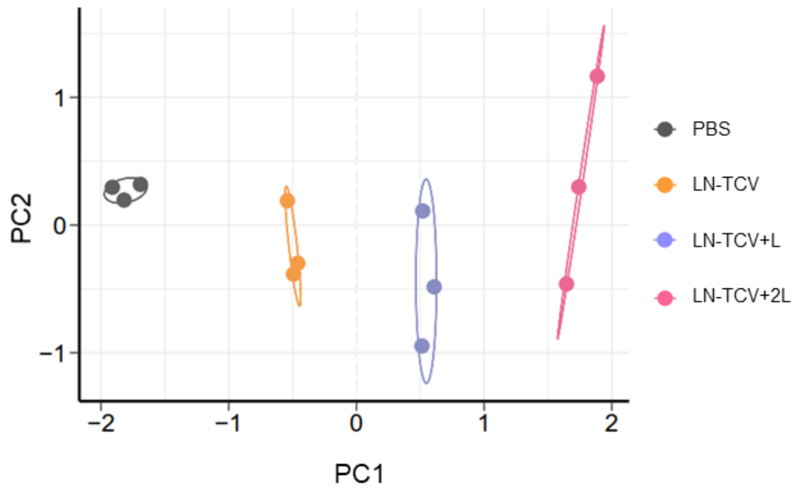

Supplementary Fig. 13. Principal component analysis plot for five indicators of immune response in lymph nodes derived from data in Fig. 3 (n = 3 mice per group). Each dot represents an animal, the color of dots denotes the group, and the ellipses show the distribution of groups as 68% confidence levels assuming a multivariate normal distribution. Upon this dimension reduction process, the data source from four groups could be clearly represented as four corresponding clusters, showing the substantial discrepancy between different treatments.

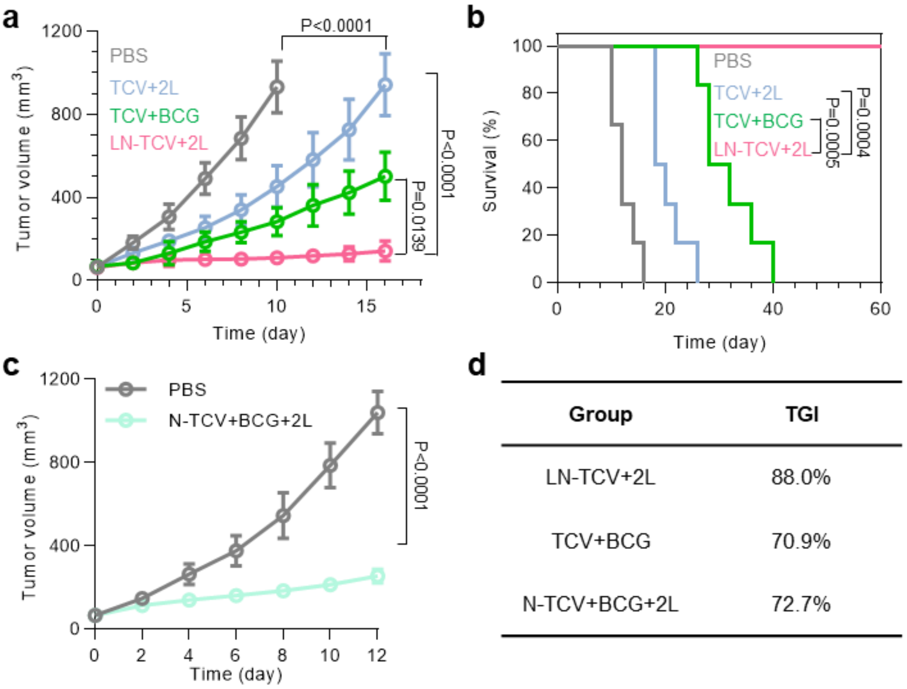

Supplementary Fig. 14. Evaluation of therapeutic effect in various treatments against primary 4T1 model.

(a) Tumor growth curves of different treatments (PBS, TCV+2L, TCV+BCG, and LN-TCV+2L). The P values of TCV +2L to PBS, TCV+BCG to TCV+2L, and LN-TCV+2L to TCV+BCG were <0.0001, <0.0001, and 0.0139, respectively.

(b) The survival curves of the mice treated with indicated groups (n = 6 mice per group). The 4T1 tumor-bearing mice treated with TCV+2L had a moderate tumor growth inhibition, demonstrating that photothermal NPs loaded in TCV were indispensable for on-demand antitumor therapy and consequent potent therapeutic outcomes. Furthermore, owing to the adjuvanticity of BCG, the antitumor efficacy was further improved in TCV+BCG group. In addition, treating tumor-bearing mice with LN-TCV+2L nearly completely inhibited tumor growth with a 100% survival rate after 60 days, due to the on-demand NIR manipulation of immune response. The P values of LN-TCV+2L to TCV+2L, and LN-TCV+2L to TCV+BCG were 0.0004 and 0.0005.

(c) Average tumor growth curves in PBS and N-TCV+BCG+2L groups. The P value of N-TCV+BCG+2L to PBS was <0.0001.

(d) TGI comparison. Note that the TGI value of LN-TCV+2L and TCV+BCG was calculated from the Data in Supplementary Fig. 14a. Moderated tumor inhibition effect was observed in the mice receiving N-TCV+BCG+2L, with the TGI value of 72.7%. On the contrary, LN-TCV+2L significantly delayed the tumor development, with the TGI value up to 88.0%, further supporting our rational design and strong therapeutic benefits. TGI value was calculated by the average tumor size of six mice per group.

Data in a and c were represented the means ± s.d., n = 6 mice per group.

P values in a were calculated by using one-way ANOVA.

P values in b were determined using log-rank test.

P value in c were calculated by two-tailed unpaired Student's t-test.

a

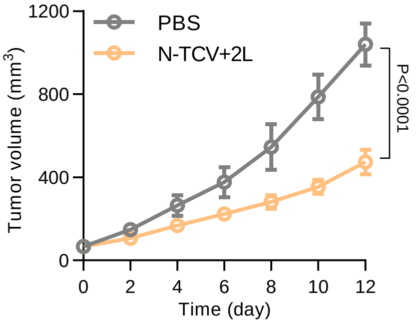

b

Group	TGI
LN-TCV+2L	90.2%
N-TCV+2L	55.1%

Supplementary Fig. 15. Evaluation of therapeutic effect in N-TCV+2L against primary 4T1 model.

(a) Average tumor growth curves in PBS and N-TCV+2L groups. The P value of N-TCV+2L to PBS was <0.0001.

(b) TGI comparison of N-TCV +2L and LN-TCV+2L at day 10. Note that the TGI value of LN-TCV+2L was calculated from the data in Fig. 4b. The mice receiving LN-TCV+2L showed significantly delayed tumor development (TGI = 90.2%), while that in N-TCV+2L group exhibited a slight tumor inhibition (TGI = 55.1%) due to the lack of HSPs. TGI value was calculated by the average tumor size of six mice per group.

Data in a were represented the means ± s.d., n = 6 mice per group.

P value in a were calculated by using two-tailed unpaired Student's t-test.

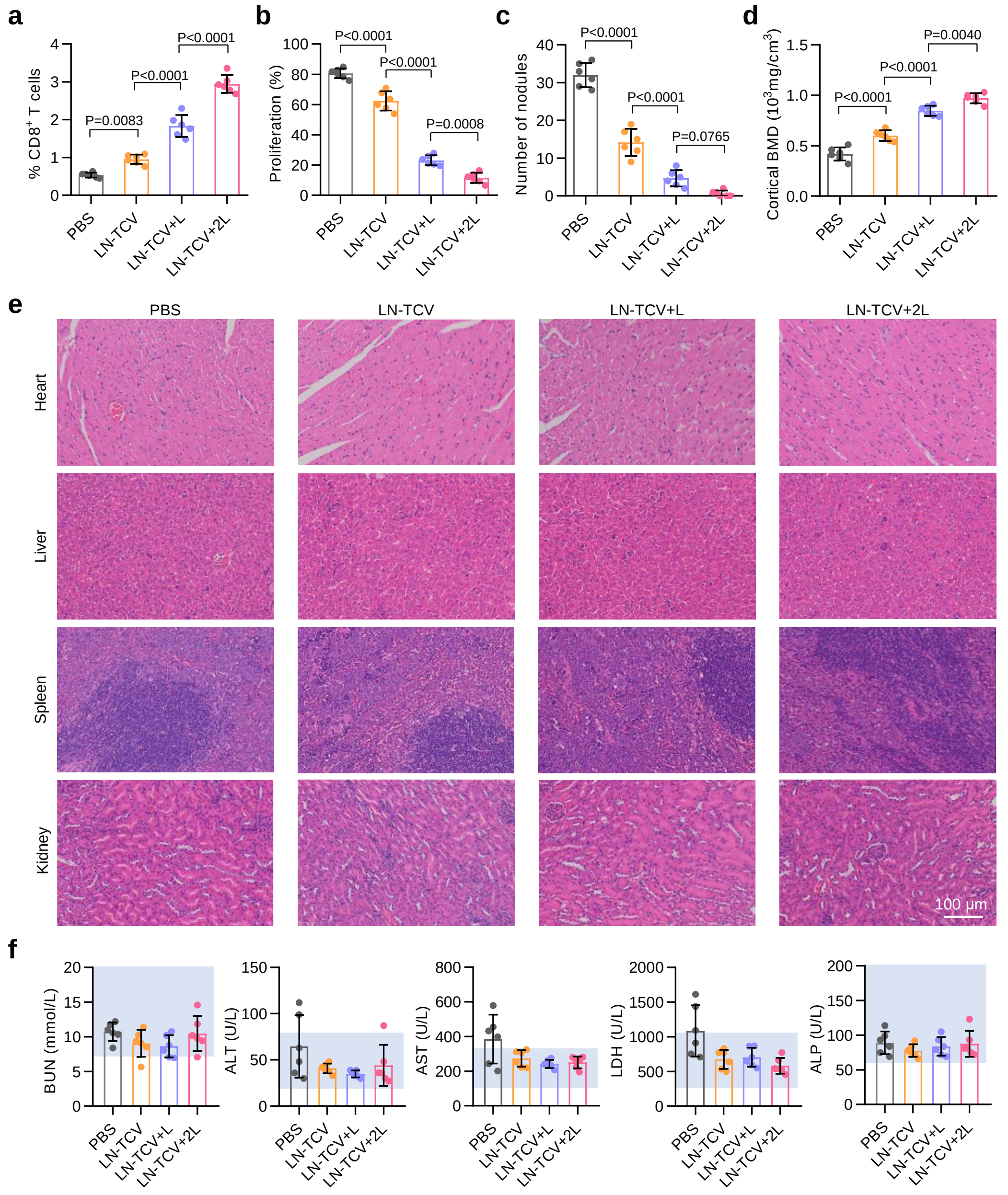

Supplementary Fig. 16. Supplementary data of LN-TCV-based treatments in primary 4T1 model.

(a) The CD8$^+$ T cells infiltration in different groups (PBS, LN-TCV, LN-TCV+L, and LN-TCV+2L). After twice NIR laser irradiation on vaccination site, more CD8$^+$ T cells infiltrated into tumor compared to those in other groups, which indicated that this strategy could effectively elicit immune response for inhibiting tumor growth. The P values of LN-TCV to PBS, LN-TCV+L to LN-TCV, and LN-TCV+2L to LN-TCV+L were 0.0083, <0.0001, and <0.0001, respectively.

(b) The proliferation rates of tumors in indicated groups. After twice NIR laser

irradiation to injection site, tumor cells were almost killed, indicating the effective tumor growth suppression. The P values of LN-TCV to PBS, LN-TCV+L to LN-TCV, and LN-TCV+2L to LN-TCV+L were <0.0001, <0.0001, and 0.0008, respectively.

(c) Quantitative analysis for lung metastasis in indicated groups. The P values of LN-TCV to PBS, LN-TCV+L to LN-TCV, and LN-TCV+2L to LN-TCV+L were <0.0001, <0.0001, and 0.0765, respectively.

(d) Quantitative analysis for bone metastases in indicated groups. After twice NIR laser irradiation on vaccination site, tumor metastasized to the lungs and bones were effectively suppressed. The P values of LN-TCV to PBS, LN-TCV+L to LN-TCV, and LN-TCV+2L to LN-TCV+L were <0.0001, <0.0001, and 0.0040, respectively.

(e) Hematoxylin-eosin(H&E)-stained tissue sections in indicated groups. There were few abnormalities in the main organs, confirming the safety of our LN-TCV platform. The images were representative of six independent mice and were presented with same magnification.

(f) Serum biochemical parameters in indicated groups. All the markers including aspartate aminotransferase (AST), alanine aminotransferase (ALT), blood urea nitrogen (BUN), lactic dehydrogenase (LDH), and alkaline phosphatase (ALP) were within normal ranges (blue areas) in vaccination groups, which demonstrated the safety of LN-TCV platform.

Data a, b, c, d, and f were represented as mean values ± s.d., n = 6 mice per group.

P values in a, b, c, and d were calculated by using one-way ANOVA.

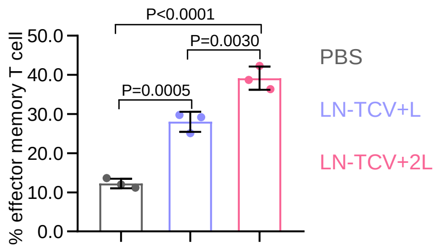

Supplementary Fig. 17. Supplementary data of quantification of effector memory T cells in the spleen. The P values of LN-TCV+L to PBS, LN-TCV+2L to LN-TCV+L, and LN-TCV+2L to PBS were 0.0005, 0.0030, and <0.0001, respectively.

Data were represented as mean values ± s.d., n = 3 mice per group.

P values were calculated by using one-way ANOVA.

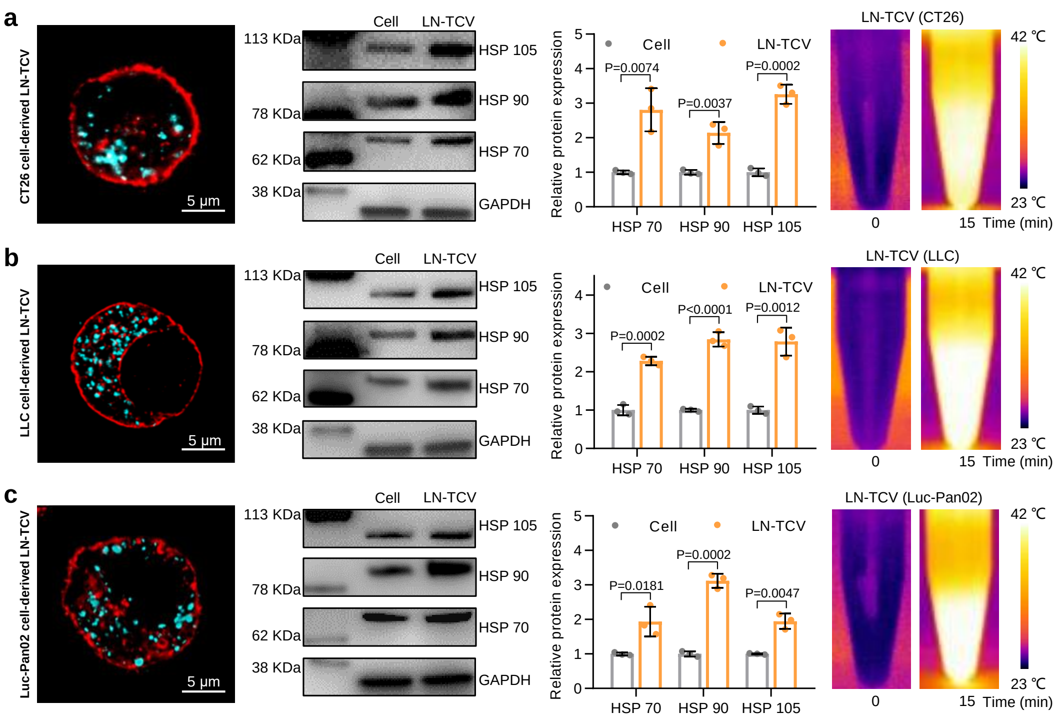

Supplementary Fig. 18. Universality of the construction method for LN-TCV with various cell lines.

The construction method was applied to other types of tumor cells (CT26, LLC, and Luc-Pan02). CT26 cell-based LN-TCV (a), LLC cell-based LN-TCV (b), and Luc-Pan02 cell-based LN-TCV (c) all possessed intact cell membranes, high level of HSPs (HSP 70, HSP 90, and HSP 105) expression, and great photothermal property. Red: cell membrane; Cyan: NPs. The P values of LN-TCV to Cell were 0.0074 for the relative protein expression of HSP 70, 0.0037 for the relative protein expression of HSP 90, and 0.0002 for the relative protein expression of HSP 105 in a. The P values of LN-TCV to Cell were 0.0002 for the relative protein expression of HSP 70, <0.0001 for the relative protein expression of HSP 90, and 0.0012 for the relative protein expression of HSP 105 in b. The P values of LN-TCV to Cell were 0.0181 for the relative protein expression of HSP 70, 0.0002 for the relative protein expression of HSP 90, and 0.0047 for the relative protein expression of HSP 105 in c.

Quantitative data were represented as mean values ± s.d., n = 3 biologically independent samples.

P values were calculated by using two-tailed unpaired Student's t-test.

The experiments in a-c were repeated three times independently with similar results.

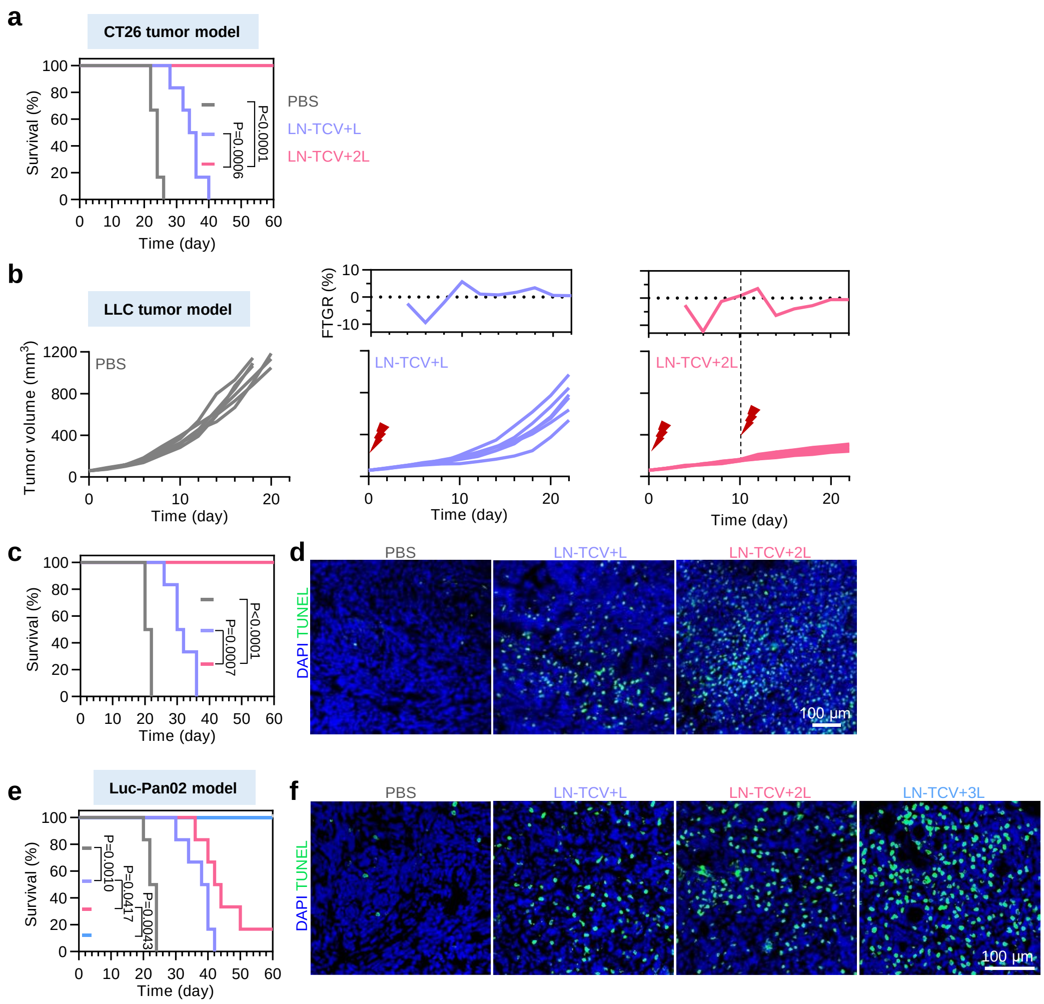

Supplementary Fig. 19. Therapeutic effect of various treatments in different tumor models (CT26, LLC, and Luc-Pan02).

(a) Overall survival curves of CT26 tumor model in different groups (PBS, LN-TCV+L, and LN-TCV+2L) (n = 6 mice per group). The P values of LN-TCV+2L to PBS and LN-TCV+2L to LN-TCV+L were <0.0001 and 0.0006.

(b) LLC tumor growth curves and the corresponding FTGR values analysis in different groups (PBS, LN-TCV+L, and LN-TCV+2L) (n = 6 mice per group). The mice were vaccinated with LN-TCV and irradiated with NIR laser irradiation (808 nm, 0.65 W/cm^2, 20 min) at day 0. When the FTGR value exceeded 0 at day 10, the NIR laser irradiation was given to the mice for achieving on-demand therapy.

(c) Overall survival curves of LLC tumor model in indicated groups (n = 6 mice per group). The P values of LN-TCV+2L to PBS and LN-TCV+2L to LN-TCV+L were <0.0001 and 0.0007.

(d) **The TUNEL analysis in LLC tumors with indicated treatments.** As a large amount of $CD8^{+}$T cells infiltrated into LLC tumor, tumor cells were effectively killed, which indicated that tumor could be effectively suppressed by increasing the number of light exposures. The images were representative of three independent mice and were presented with same magnification.

(e) **Overall survival curves of Luc-Pan02 tumor model in different groups (PBS, LN-TCV+L, LN-TCV+2L, and LN-TCV+3L) (n = 6 mice per group).** The P values of LN-TCV+L to PBS, LN-TCV+2L to LN-TCV+L, and LN-TCV+3L to LN-TCV+2L were 0.0010, 0.0417, and 0.0043, respectively.

(f) **The TUNEL analysis in Luc-Pan02 tumors with indicated groups.** As a large amount of $CD8^{+}$ T cells infiltrated into Luc-Pan02 tumor, tumor cells were effectively killed, which indicated that more malignant tumor could also be effectively suppressed by increasing the times of NIR laser irradiation. The images were representative of three independent mice and were presented with same magnification.

P values in a, c, and e were calculated by using the log-rank test.

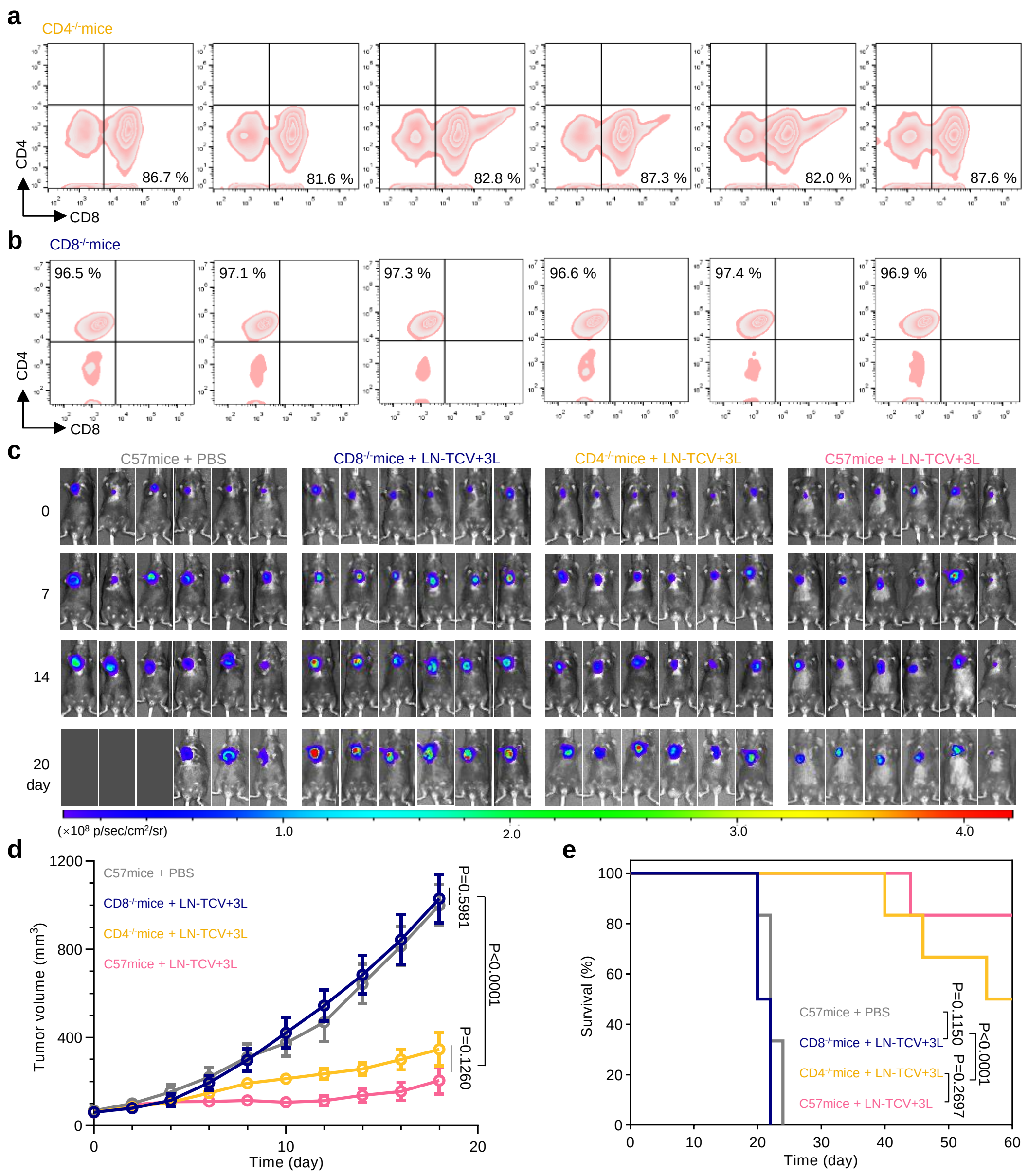

Supplementary Fig. 20. Evaluations of CD8$^+$ T cell-specific antitumor effects in the Luc-Pan02 tumor model.

(a) FCM plots for CD8$^+$ T and CD4$^+$ T cells in CD4-KO (CD4$^{-/-}$) mice (n = 6 mice per group). These results implied that the mice we used were completely knocked out for CD4$^+$ T cells.

(b) FCM plots for CD8$^+$ T and CD4$^+$ T cells in CD8-KO (CD8$^{-/-}$) mice (n = 6 mice per group). These results implied that the mice we used were completely knocked out for CD8$^+$ T cells.

(c) In vivo bioluminescence images of antitumor effects in various mice (C57, CD4$^{-/-}$, and CD8$^{-/-}$) treated with LN-TCV (n = 6 mice per group).

(d) **Tumor growth curves of different treatments (C57 Mice + PBS, C57 Mice + LN-TCV+3L, CD4$^{-/-}$Mice + LN-TCV+3L, and CD8$^{-/-}$ Mice + LN-TCV+3L) (n = 6 mice per group).** The P values of CD8$^{-/-}$ Mice + LN-TCV+3L to PBS, C57 Mice + LN-TCV+3L to CD4$^{-/-}$Mice + LN-TCV+3L, and C57 Mice + LN-TCV+3L to CD8$^{-/-}$ Mice + LN-TCV+3L were 0.5981, 0.1260, and <0.0001, respectively.

(e) **The survival curves of tumor-bearing mice treated with different groups (n = 6 mice per group).** The P values of CD8$^{-/-}$ Mice + LN-TCV+3L to PBS, C57 Mice + LN-TCV+3L to CD4$^{-/-}$Mice + LN-TCV+3L, and C57 Mice + LN-TCV+3L to CD8$^{-/-}$ Mice + LN-TCV+3L were 0.1150, 0.2697, and <0.0001, respectively.

Compared with wild type mice (C57), the CD8 knockout mice (CD8$^{-/-}$) received same treatment exhibited significantly compromised effect on inhibiting tumor growth and prolonging survival time, thus demonstrating the importance of CD8^{+}T cells in antitumor effect.

Data in d were represented as mean values ± s.d..

P values in d were calculated by using one-way ANOVA.

P values in e were calculated by using log-rank test.

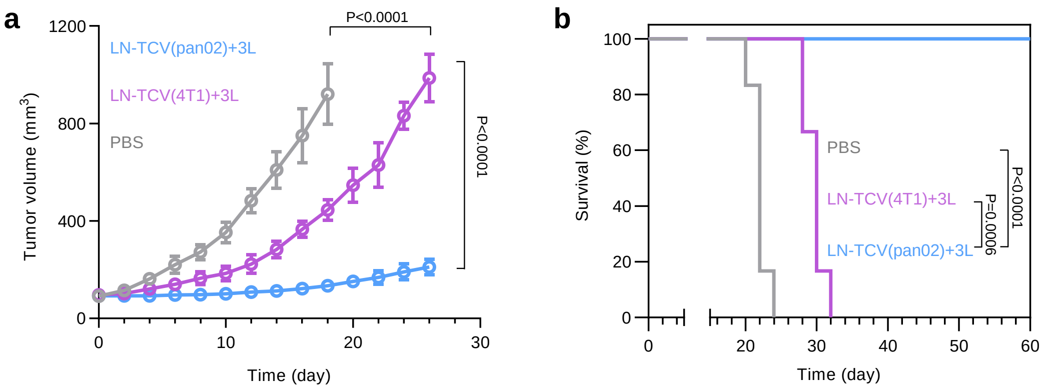

Supplementary Fig. 21. The specificity of LN-TCV in Luc-Pan02 tumor model.

(a) Tumor growth curves of different treatments (PBS, LN-TCV(4T1)+3L, and LN-TCV(Pan02)+3L). The P values of LN-TCV(4T1)+3L to PBS and LN-TCV(Pan02)+3L to LN-TCV(4T1)+3L were both <0.0001.

(b) The survival curves of the mice treat with indicated groups (n = 6 mice per group). The P values of LN-TCV(Pan02)+3L to PBS and LN-TCV(Pan02)+3L to LN-TCV(4T1)+3L were <0.0001 and 0.0006.

Compared with LN-TCV(4T1), the LN-TCV(Pan02) could effectively inhibit Luc-Pan02 tumor growth and prolong the survival time upon NIR irradiation, indicating that the specificity of the vaccine.

Data in a were represented as mean values ± s.d, n = 6 mice per group.

P values in a were calculated by using one-way ANOVA.

P values in b were calculated by using log-rank test.

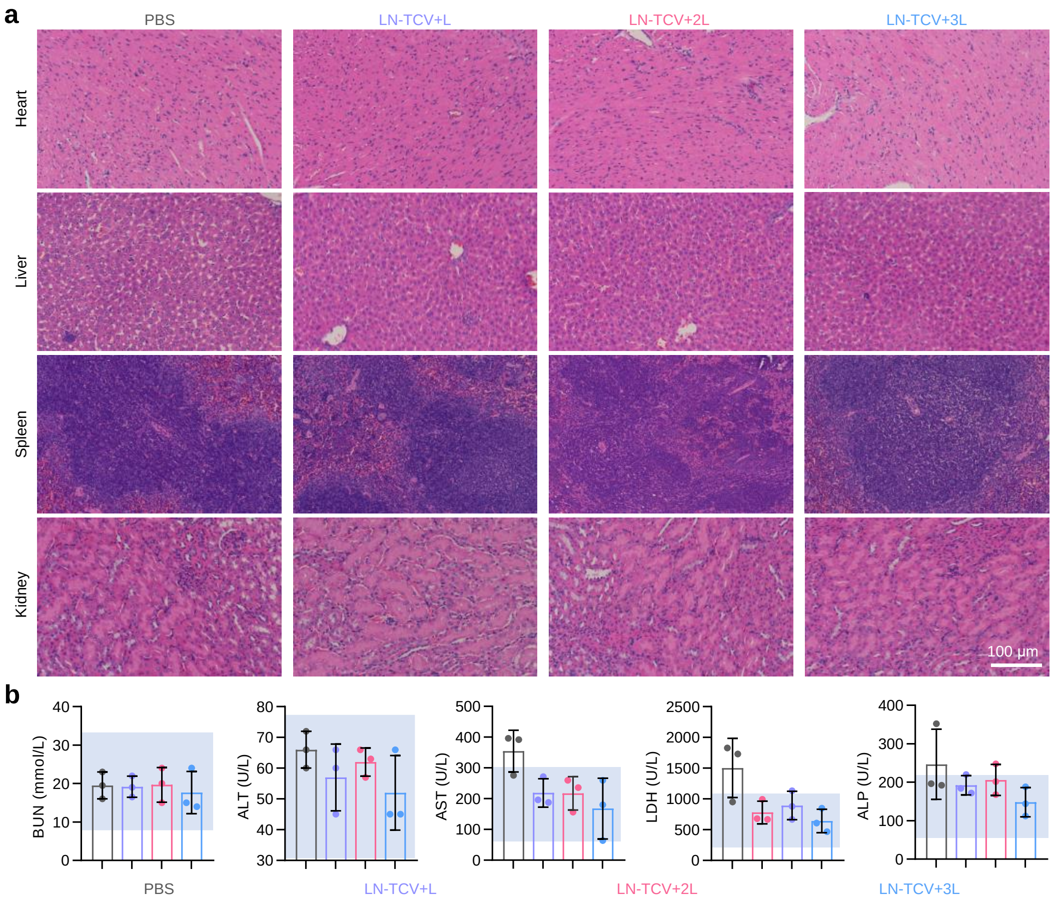

Supplementary Fig. 22. Biosafety evaluation of LN-TCV based treatments in primary Luc-Pan02 model.

(a) H&E-stained tissue sections in different groups (PBS, LN-TCV+L, LN-TCV+2L, and LN-TCV+3L). There were few abnormalities in the main organs, confirming the safety of our LN-TCV platform. The images were representative of three independent mice and were presented with same magnification.

(b) Serum biochemical parameters in indicated treatments. All the markers including AST, ALT, BUN, LDH, and ALP were within normal ranges (blue areas), which demonstrated the safety of vaccination with different times of NIR laser irradiations.

Data in b were represented as mean values ± s.d., n = 3 mice per group.

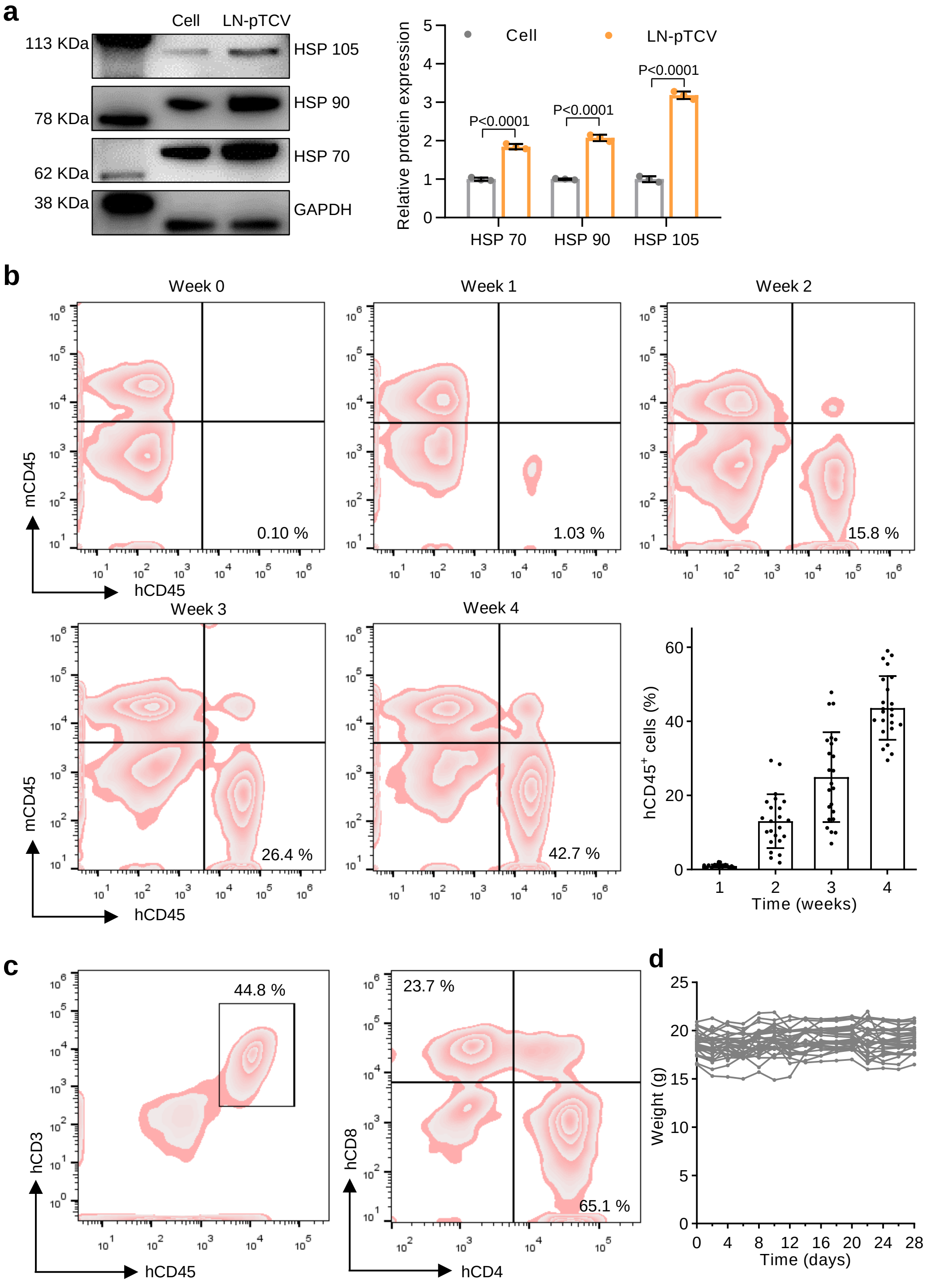

Supplementary Fig. 23. Reconstitution of human immunity in NTG recipient mice.

(a) Western blotting analysis and quantification for the expression of HSP 70, HSP 90, and HSP 105 in patient-derived tumor cells and LN-pTCV (n = 3 biologically independent samples). The concept of LN-TCV also could extend to patient tumor cells for constructing LN-pTCV with an increased expression of HSPs.

The P values of LN-TCV to Cell for the relative protein expression of HSP 70, HSP 90, and HSP105 were <0.0001.

(b) Representative FCM plots and quantitative analysis for human CD45$^+$ (hCD45$^+$) cells in the peripheral blood of humanized mice per week after the intravenous injection of human peripheral blood mononuclear cells (PBMCs) (n = 23 mice). Increased percentages of hCD45$^+$ cells were found in peripheral blood over time. The percent of peripheral blood hCD45$^+$ cells in each mouse were greater than 25%, indicating the successful construction of humanized mice. Thus, all of the mice were successfully reconstituted human immunity after 4 weeks.

(c) Representative FCM plots of T cell marker expression in peripheral blood of humanized mice. High levels of human cell engraftment were found in peripheral blood. Engrafted hCD45$^+$ cells mainly consisted of hCD3$^+$ T cells. These data confirmed the successful reconstitution of human immunity in NTG recipients. The images were representative of twenty-three independent mice.

(d) Body weight change curves of mice in the progress of humanization (n = 23 mice).

Quantitative data in a and b were presented as mean values ± s.d..

P values in a were calculated by using two-tailed unpaired Student’s t-test.

The experiment in a was repeated three times independently with similar results.

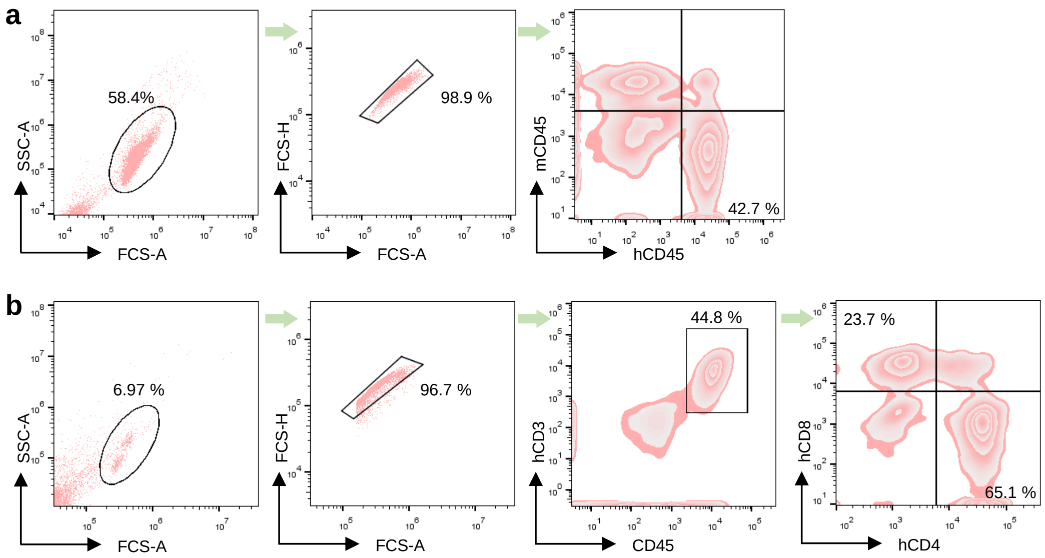

Supplementary Fig. 24. Flow cytometry gating strategies for Supplementary Fig. 23.

(a) Flow cytometry gating strategies for hCD45$^+$ cells in the peripheral blood of humanized mice (related to supplementary Fig. 23b).

(b) Flow cytometry gating strategies for T cell marker expression in peripheral blood of humanized mice (related to supplementary Fig. 23c).

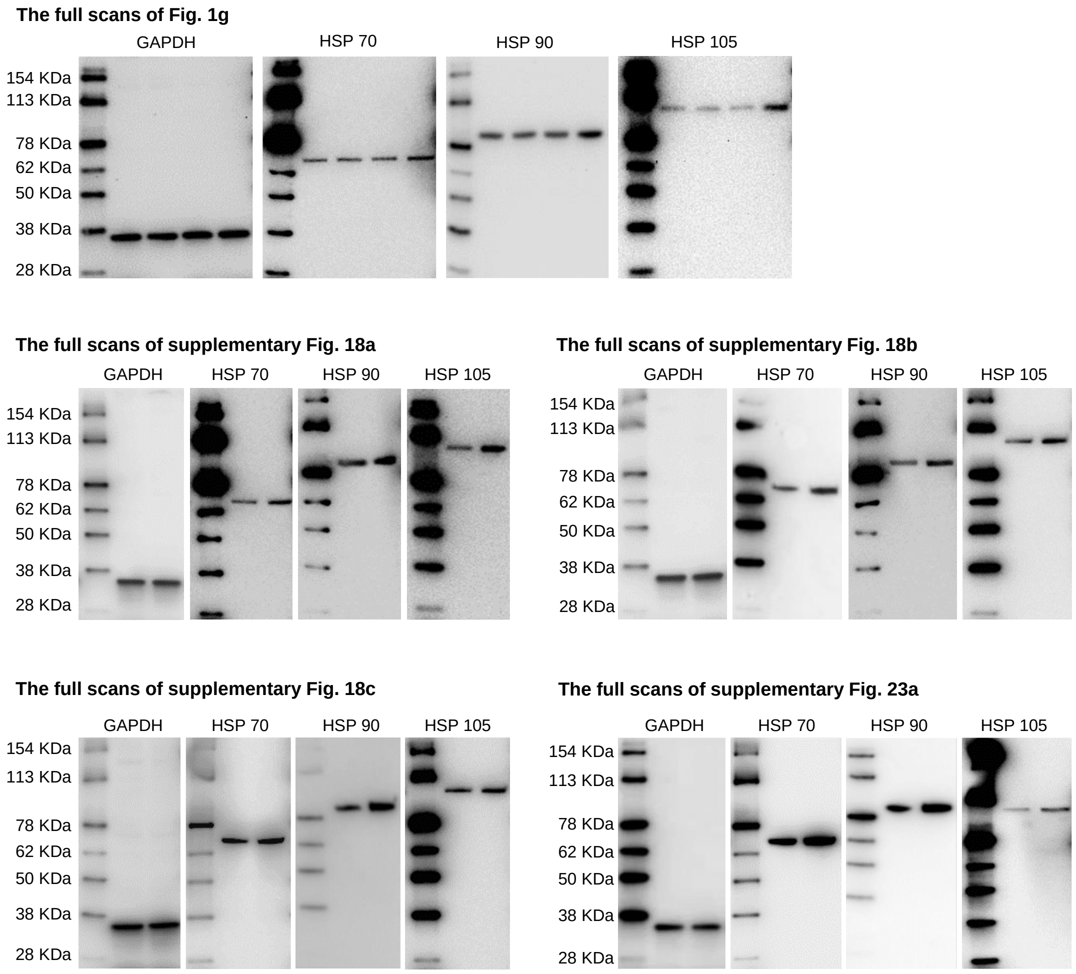

Supplementary Fig. 25. Full-length western blotting images.

Supplementary Table. 1 List of antibodies used for flow cytometry in this study.

Antibodies	Source	Identifier	Clone	Dilution
PE anti-mouse CD11c	Biolegend	cat.no. 117308	N418	1:50
APC anti-mouse CD80	Biolegend	cat.no. 104714	16-10A1	1:50
APC/Cyanine7 anti-mouse CD86	Biolegend	cat.no. 105030	GL-1	1:50
FITC anti-mouse CD11c	Biolegend	cat.no. 117305	N418	1:50
PerCP/Cyanine5.5 anti-mouse CD86	Biolegend	cat.no. 105027	GL-1	1:50
PE/Cyanine7 anti-mouse CD80	Biolegend	cat.no. 104733	16-10A1	1:50
APC anti-mouse CD40	Biolegend	cat.no. 124611	3/23	1:50
APC anti-mouse CD4	Biolegend	cat.no. 100411	GK1.5	1:50
PE anti-mouse CD3	Biolegend	cat.no. 100206	17A2	1:50
Brilliant Violet 605 anti-mouse CD8a	Biolegend	cat.no. 100743	53-6.7	1:50
PE anti-human CD45	Biolegend	cat.no. 304008	HI30	1:50
Alexa Flour647 anti-mouse granzyme B	Biolegend	cat.no. 515405	GB11	1:50
FITC anti-mouse CD45	Biolegend	cat.no. 157214	S18009F	1:50
APC anti-mouse CD62L	Biolegend	cat.no. 104411	MEL-14	1:50
Brilliant Violet 605 anti-human CD3	Biolegend	cat.no. 317322	OKT3	1:50
PerCP/Cyanine5.5 anti-mouse IFN-γ	eBioscience	cat.no. 45-7311-82	XMG1.2	1:50
eflour450 anti-mouse CD44	eBioscience	cat.no. 48-0441-82	IM7	1:50
PerCP/eFlour 710 anti-mouse MHC Class I (H-2Kd)	eBioscience	cat.no. 46-5957-82	SF1-1.1.1	1:50
PE- Cyanine7 anti-human CD4	eBioscience	cat.no. 25-0049-42	RPA-T4	1:50
Alexa Flour700 anti-human CD8	eBioscience	cat.no. 56-0088-42	RPA-T8	1:50
Purified Rat Anti-Mouse CD16/CD32	BD	cat.no. 553141	2.4G2	1:50
Ghost Dye UV450	Tonbo	cat.no. 13-0863		1:500

Supplementary Table. 2 List of antibodies used in this study.

Antibodies	Source	Identifier	Clone	Dilution
Anti-GAPDH Antibody	Affinity	cat.no. AF7021	Polyclonal	1:3000
Anti-HSP70 Antibody	Abcam	cat.no. ab194360	EPR16893	1:5000
Anti-HSP90 Antibody	Abcam	cat.no. ab87133	polyclonal	1:5000
Anti-HSP105 Antibody	Abcam	cat.no. ab109624	EPR4576	1:5000
Goat Anti-Rabbit IgG (H+L) HRP	Affinity	cat.no. S0001	Polyclonal	1:2000
Anti-CD11c Antibody	Abcam	cat.no. ab254183	KB90	1:100
Anti-CD8 Antibody	Invitrogen	cat.no. 14-0081-82	53-6.7	1:50
Anti-Ki67 Antibody	Abcam	cat.no. ab16667	SP6	1:100
Anti-Granzyme B Antibody	Abcam	cat.no. ab255598	EPR22645-206	1:200
Anti-Calreticulin Antibody, Alexa Fluor 488	Abcam	cat.no. ab196158	EPR3924	1:1000
Anti-HMGB1 Antibody	Abcam	cat.no. ab79823	EPR3507	1:1000
Goat anti-Mouse IgG (H+L), Alexa Fluor Plus 647	Invitrogen	cat.no. A48265		1:1000
Donkey Anti-Rat IgG (H+L), Alexa Fluor 555	Abcam	cat.no. ab150154		1:300
Goat Anti-Rabbit IgG (H+L), FITC	Affinity	cat.no. S0008		1:300

Supplementary Table. 3 DNA sequences of mHSP promoter, mCherry, and HSP 70.

mHSP promoter	CCTGCAGCCTGAGGCAAAGGGAGTGGCTACAGCCTGGCACGGTCGATTAAGCCCTGCTCTCCGGGTCCTGGGACACTTTCCTTTTTCCTCTTTTGAGTCACAGGTCCTCCTAACATGAGAATCAAGTATTTTCACGCTGATTTCCTTATAAAATTGTGAGAACTCCATAGGCGATGTACCGCCTACTCCTACCTTAACCGTGATGTAAAGACAGCAAAACAAATGAACTATACTGCAAGATCTCTTCTATTTCCCTATTCAAACCTAAAATGAAGAGGGAGGGGGAGACATGGACAAGCAAGCATTCCACAGGCGCCCCTGCCCAACGCTGTCACTCAAACCAGGACCCAATCACAGACTTTTTAGCCAAGCCTTATCCCGCCTCTCTTGAGAAACTTTCTGCGTCCGCCATCCTGTAGGAAGGATTTGTACACTTTAAACTCCCTCCCTGGTCTGAGTCCCACACTCTCACCACCCAGCACCTTCAGGAGCTGACCCTTAACAGCTTCACCCACAGGGACCCCGAAGTTGCGTCGCCTCCGCAACAGTGTCAATAGCAGCACCAGCACTTCCCCACACCCTCCCCCTCAGGAATCCGTACTCTCTAGCGAACCCCAGAAACCTCTGGAGAGTTCTGGACAAGGGCGGAACCCACAACTCCGATTACTCAAGGGAGGCGGGGAAGCTCCACCAGACGCGAAACTGCTGGAAGATTCCTGGCCCCAAGGCCTCCTCCGGCTCGCTGATTGGCCCAGCGGAGAGTGGGCGGGGCCGGTGAAGACTCCTTAAAGGCGCAGGGCGGCGAGCAGGGCACCAGACGCTGACAGCTACTCAGAATCAAATCTGGTTCCATCCAGAGACAAGCGAAGACAAGAGAAGCAGAGCGAGCGGCGCGTTCCCGATCCTCGGCCAGGACCAGCCTTCCCCAGAGCATCCACGCCGCGGAGCGCAACCTTCCCAGGAGCATCCCTGCCGCGGAGCGCAACTTTCCCCGGAGCATCCACGCCGCGGAGCGCAGCCTTCCAGAAGCAGAGCGCGGCGCCACC
mCherry	ATGGTGTCTAAAGGGGAGGAAGACAACATGGCAATCATTAAGGAATTTATGCGCTTCAAAGTGCACATGGAGGGATCTGTGAATGGACATGAGTTTGAGATAGAAGGGGAGGGTGAAGGGAGGCCTTATGAGGGTACTCAGACTGCAAAGCTGAAGGTAACCAAGGGCGGACCCCTGCCCTTTGCATGGGATATACTCTCTCCTCAGTTCATGTATGGCTCCAAGGCTTATGTGAAACACCCCGCTGACATCCCTGACTACCTAAAGCTCAGTTTCCCTGAAGGTTTTAAGTGGGAAAGAGTGATGAACTTTGAGGATGGGGGGGTAGTTACGGTGACTCAAGACAGTTCATTGCAAGATGGCGAGTTCATCTACAAAGTAAAACTACGAGGCACCAACTTCCCCAGCGACGGGCCTGTGATGCAGAAGAAGACTATGGGGTGGGAGGCTAGCTCAGAGCGCATGTACCCAGAGGACGGCGCCCTTAAAGGCGAGATAAAGCAGCGCCTGAAACTCAAGGATGGTGGCCACTATGATGCTGAGGTGAAGACAACCTATAAAGCCAAGAAGCCCGTCCAGCTGCCAGGGGCCTACAACGTCAACATCAAGTTGGACATCACAAGCCACAATGAAGACTATACAATTGTGGAGCAGTATGAACGAGCCGAGGGCCGCCATTCAACAGGAGGCATGGATGAACTCTACAAG
HSP 70	GGAGGCGGGGGGTCAGGCGGAGGAGGAAGTGCAAAGAACACGGCAATAGGTATAGATCTGGGTACCACCTACTCCTGTGTGGGCGTCTTCCAGCACGGGAAAGTCGAGATCATCGCTAATGACCAGGGTAACAGAACAACT

	CCAAGCTACGTGGCTTTTACAGATACTGAAAGATTAATAGGAGATGCT<br>GCAAAAAACCAGGTGGCCCTGAACCCCCAGAACACAGTGTTTGATGC<br>AAAAAGACTCATCGGGCGGAAGTTCGGAGATGCCGTGGTTCAATCTG<br>ACATGAAGCACTGGCCTTTCCAAGTGGTGAACGATGGCGACAAGCCA<br>AAGGTCCAAGTTAACTACAAGGGCGAGTCTAGATCCTTCTTCCCAGA<br>AGAGATCAGCAGCATGGTTCTTACGAAAATGAAGGAAATCGCAGAAG<br>CCTATCTGGGCCATCCAGTAACAAATGCAGTGATTACCGTGCCAGCCT<br>ACTTCAATGACTCTCAGAGGCAAGCCACCAAAGACGCCGGAGTCATT<br>GCCGGCCTCAATGTTCTGAGAATCATTAATGAGCCCACAGCTGCGGCA<br>ATTGCATACGGCTTGGACAGGACAGGCAAGGGTGAGCGAAACGTGTT<br>GATTTTCGACCTTGGAGGTGGCACATTTGATGTGAGTATCCTAACCATT<br>GACGATGGCATCTTCGAAGTGAAGGCAACAGCCGGAGACACACACCT<br>CGGAGGAGAGGACTTTGACAATCGTCTGGTTTCTCATTTTGTGGAAGA<br>GTTCAAGAGAAAGCATAAGAAGGATATCTCACAGAATAAGCGTGCAG<br>TGAGGCGTCTTCGGACTGCCTGTGAAAGGGCCAAGAGGACGTTATCT<br>TCCTCCACTCAGGCTTCACTCGAGATTGACTCGCTCTTTGAGGGAATT<br>GATTTCTACACTTCCATCACCCGGGCTCGGTTTGAAGAGCTGTGCAGT<br>GACTTATTCAGAGGGACGCTGGAACCTGTGGAGAAGGCTCTGCGCGA<br>TGCGAAGATGGATAAGGCCCAAATACATGACCTGGTCTTGGTGGGAG<br>GCTCAACCGCCATCCCGAAAGTGCAGAAACTGCTGCAGGACTTCTTT<br>AATGGGCGGGACCTGAACAAGTCAATCAACCCTGATGAAGCGGTTGC<br>CTATGGAGCTGCTGTCCAGGCCGCCATTCTGATGGGCGACAAGAGTG<br>AGAATGTACAGGATCTTCTGCTCCTGGATGTCGCCCCGCTTTCCCTCG<br>GGCTGGAAACCGCTGGCGGAGTCATGACTGCCTTAATCAAAAGGAAC<br>TCTACGATTCCAACAAAACAGACCCAAACCTTCACCACTTACTCGGAC<br>AATCAGCCAGGGGTTCTAATCCAGGTATATGAGGGCGAACGAGCCATG<br>ACCCGAGACAACAACCTCTTGGGACGGTTTGAACTATCGGGTATTCCT<br>CCTGCTCCGCGCGGTGTTCCACAGATTGAAGTAACTTTTGACATTGAT<br>GCCAACGGCATTCTAAATGTGACAGCCACAGACAAAAGCACTGGGAA<br>GGCAAATAAGATTACAATCACCAATGATAAAGGACGGCTATCCAAAGA<br>AGAAATAGAGAGGATGGTCCAAGAGGCTGAAAGGTACAAAGCTGAG<br>GACGAGGTCCAGAGAGACCGAGTAGCCGCCAAAAATGCGCTTGAGA<br>GCTATGCATTCAATATGAAAAGTGCGGTAGAGGATGAGGGGCTCAAA<br>GGTAAATTAAGCGAAGCTGATAAGAAAAAAGTTTTGGATAAGTGCCA<br>GGAGGTCATCAGCTGGCTGGATAGCAACACTCTGGCTGACAAAGAGG<br>AATTTGTTCACAAAAGGGAAGAGCTCGAGAGAGTCTGCAGCCCCATC<br>ATCAGTGGACTTTACCAGGGGGCTGGGGCTCCTGGTGCAGGAGGCTT<br>CGGAGCACAGGCGCCCAAGGGAGCGTCTGGTTCCGGCCCCACCATTG<br>AGGAGGTGGACTGA
