# Supplementary material for: Generation of whole tumor cell vaccine for on-demand manipulation of immune responses against cancer under near-infrared laser irradiation
Source: Nat Commun. 2023 Jul 26;14:4505. doi: 10.1038/s41467-023-40207-y (PMC10372023; doi:10.1038/s41467-023-40207-y)
Supplement: Supplementary file 2 — Reporting Summary [file 41467_2023_40207_MOESM2_ESM.pdf]

nature portfolio

Corresponding author(s): Zhiyuan Tian, Guanghui Ma, Wei Wei

Last updated by author(s): Jul 2, 2023

Reporting Summary

Nature Portfolio wishes to improve the reproducibility of the work that we publish. This form provides structure for consistency and transparency in reporting. For further information on Nature Portfolio policies, see our Editorial Policies and the Editorial Policy Checklist.

Statistics

For all statistical analyses, confirm that the following items are present in the figure legend, table legend, main text, or Methods section.

n/a	Confirmed	
☐	☒	The exact sample size (*n*) for each experimental group/condition, given as a discrete number and unit of measurement
☐	☒	A statement on whether measurements were taken from distinct samples or whether the same sample was measured repeatedly
☐	☒	The statistical test(s) used AND whether they are one- or two-sided<br>*Only common tests should be described solely by name; describe more complex techniques in the Methods section.*
☒	☐	A description of all covariates tested
☐	☒	A description of any assumptions or corrections, such as tests of normality and adjustment for multiple comparisons
☐	☒	A full description of the statistical parameters including central tendency (e.g. means) or other basic estimates (e.g. regression coefficient) AND variation (e.g. standard deviation) or associated estimates of uncertainty (e.g. confidence intervals)
☐	☒	For null hypothesis testing, the test statistic (e.g. *F*, *t*, *r*) with confidence intervals, effect sizes, degrees of freedom and *P* value noted<br>*Give P values as exact values whenever suitable.*
☒	☐	For Bayesian analysis, information on the choice of priors and Markov chain Monte Carlo settings
☒	☐	For hierarchical and complex designs, identification of the appropriate level for tests and full reporting of outcomes
☒	☐	Estimates of effect sizes (e.g. Cohen's *d*, Pearson's *r*), indicating how they were calculated

Our web collection on statistics for biologists contains articles on many of the points above.

Software and code

Policy information about availability of computer code

Data collection	TEM images were obtained using HT7000 microscope (Hitachi, Japan). The size distribution and zeta potential were determined using Malvern laser particle size analyzer (Malvern ZEN 3600 Zetasizer, UK) with associated software (NANO ZS, version 7.12). The temperatures were recorded by an infrared thermal camera (FLIR E40, USA) and treated with associated software (FLIR Tools, version 4.0.13330.1003). The images of immunohistochemistry (IHC) and immunofluorescence (IF) were visualized by using an automatic multispectral imaging system with associated software (Vectra II, PerkinElmer, version 2.0.7.1). The images of western blotting were obtained using a Multicolor fluorescent gel imaging system with associated software (DNR MF ChemiBIS3.2, Israel, version 7.0.12). Fluorescent images were collected using CLSM (Nikon A1, Japan) with associated software (NIS-Elements AR 5.20.00). Flow cytometry data was acquired with Backman CytoFLEX LX Flow Cytometer with associated software (version 2.3.1.22). The mice fluorescence images were observed by using animal imaging systems with associated softwares (Kodak FX Pro, Japan, version 5.4.2.18893 and PerkinElmer, USA, version 4.5.5).the serum biochemistry data were obtained by using an automated analyzer (Hitachi Ltd Hitachi-917, Japan).
Data analysis	Statistical analyses were carried out via GraphPad Prism 9.0.0. RNAs were identified using the fold-change method with the R package DEGseq (R-3.6.2). Fluorescent images were analyzed by ImageJ 1.8.0.

For manuscripts utilizing custom algorithms or software that are central to the research but not yet described in published literature, software must be made available to editors and reviewers. We strongly encourage code deposition in a community repository (e.g. GitHub). See the Nature Portfolio guidelines for submitting code & software for further information.

Data

Policy information about availability of data

All manuscripts must include a data availability statement. This statement should provide the following information, where applicable:

- Accession codes, unique identifiers, or web links for publicly available datasets
- A description of any restrictions on data availability
- For clinical datasets or third party data, please ensure that the statement adheres to our policy

The main data supporting the results in this study are available within the paper and its Supplementary Information. Source data for the figures in the main text are available at Figshare (https://doi.org/10.6084/m9.figshare.23617533.v1). Source data for the figures in the Supplementary Information are available at Figshare (https://doi.org/10.6084/m9.figshare.23617539.v2). The RNA-seq has been deposited to NCBI under accession number (SRP390019).

Human research participants

Policy information about studies involving human research participants and Sex and Gender in Research.

Reporting on sex and gender	The pancreatic tumor samples for PDX model were obtained from a female patient with pancreatic carcinoma.
Population characteristics	Resected pancreatic tumor samples for PDX model construction were obtained from a pancreatic carcinoma patient in China-Japan Union Hospital of Jilin University. The patient population covered pancreatic carcinoma (female, primary pancreatic carcinoma without metastasis).
Recruitment	Tumor samples obtained from pancreatic carcinoma patient with informed consent from China-Japan Union Hospital of Jilin University in accordance with the guidelines given by the ethical review board.
Ethics oversight	PDX studies were approved by the Scientific Research Ethical Committee of China-Japan Union Hospital of Jilin University for Human Biomedical Research (2021080201) and informed consent was obtained from the participant.

Note that full information on the approval of the study protocol must also be provided in the manuscript.

Field-specific reporting

Please select the one below that is the best fit for your research. If you are not sure, read the appropriate sections before making your selection.

☒ Life sciences ☐ Behavioural & social sciences ☐ Ecological, evolutionary & environmental sciences

For a reference copy of the document with all sections, see nature.com/documents/nr-reporting-summary-flat.pdf

Life sciences study design

All studies must disclose on these points even when the disclosure is negative.

Sample size	In this study, sample size was determined by referring to pilot studies and relevant literature. For in vitro and ex vivo experiments, we used at least 3 biological replicates per group. For the experiments for tumor inhibition, the sample size was 6 independently mice each group.
Data exclusions	No data were excluded.
Replication	Experiments were replicated multiple times and obtained the similar results. For in vitro studies, experiments were replicated in 3 biological independent samples. For tumor inhibition studies, experiments were replicated twice and the data of one representative experiment with 6 independently mice were shown.
Randomization	For the in vitro experiments, samples were randomly allocated into experimental groups. For the in vivo studies, animals were randomly grouped.
Blinding	During data collection and analysis for the experiments, investigators were blinded to group allocation.

Reporting for specific materials, systems and methods

We require information from authors about some types of materials, experimental systems and methods used in many studies. Here, indicate whether each material, system or method listed is relevant to your study. If you are not sure if a list item applies to your research, read the appropriate section before selecting a response.

Materials & experimental systems

n/a	Involved in the study
	x Antibodies
	x Eukaryotic cell lines
x	Palaeontology and archaeology
	x Animals and other organisms
x	Clinical data
x	Dual use research of concern

Methods

n/a	Involved in the study
x	ChIP-seq
	x Flow cytometry
x	MRI-based neuroimaging

Antibodies

Antibodies used

Flow Cytometry:
1. PE anti-mouse CD11c Antibody (Biolegend, cat.no.117308, clone: N418, 1:50)
2. APC anti-mouse CD80 Antibody (Biolegend, cat.no.104714, clone: 16-10A1, 1:50)
3. APC/Cyanine7 anti-mouse CD86 Antibody (Biolegend, cat.no. 105030, clone: GL-1, 1:50)
4. PerCP/eFlour 710 anti-mouse MHC Class I (H-2Kd) Antibody (eBioscience, cat.no. 46-5957-82, clone: SF1-1.1.1, 1:50)
5. FITC anti-mouse CD11c Antibody (Biolegend, cat.no.117305, clone: N418, 1:50)
6. PerCP/Cyanine5.5 anti-mouse CD86 Antibody (Biolegend, cat.no. 105027, clone: GL-1, 1:50)
7. PE/Cyanine7 anti-mouse CD80 Antibody (Biolegend, cat.no. 104733, clone: 16-10A1, 1:50)
8. APC anti-mouse CD40 Antibody (Biolegend, cat.no. 124611, clone: 3/23, 1:50)
9. APC anti-mouse CD4 Antibody (Biolegend, cat.no. 100411, clone: GK1.5, 1:50)
10. PE anti-mouse CD3 Antibody (Biolegend, cat.no. 100206, clone: 17A2, 1:50)
11. Brilliant Violet 605 anti-mouse CD8a Antibody (Biolegend, cat.no. 100743, clone: 53-6.7, 1:50)
12. PerCP/Cyanine5.5 anti-mouse IFN-γ Antibody (eBioscience, cat.no. 45-7311-82, clone: XMG1.2, 1:50)
13. Alexa Flour647 anti-mouse granzyme B Antibody (Biolegend, cat.no. 515405, clone: GB11, 1:50)
14. eflour450 anti-mouse CD44 Antibody (eBioscience, cat.no. 48-0441-82, clone: IM7, 1:50)
15. APC anti-mouse CD62L Antibody (Biolegend, cat.no. 104411, clone: MEL-14, 1:50)
16. FITC anti-mouse CD45 Antibody (Biolegend, cat.no. 157214, clone: S18009F, 1:50)
17. PE anti-human CD45 Antibody (Biolegend, cat.no. 304008, clone: HI30, 1:50)
18. Brilliant Violet 605 anti-human CD3 Antibody (Biolegend, cat.no. 317322, clone: OKT3, 1:50)
19. PE- Cyanine7 anti-human CD4 Antibody (eBioscience, cat.no. 25-0049-42, clone: RPA-T4, 1:50)
20. Alexa Flour700 anti-human CD8 Antibody (eBioscience, cat.no. 56-0088-42, clone: RPA-T8, 1:50)
21. Purified Rat Anti-Mouse CD16/CD32 (Mouse BD Fc Block™) (BD, cat.no. 553141, clone: 2.4G2, 1:50)
22. Ghost Dye UV450 (Tonbo, cat.no. 13-0863, 1:500)

Western blotting:
23. anti-GAPDH Antibody (Affinity, cat.no. AF7021, clone: Polyclonal, 1:3000)
24. anti-HSP70 Antibody (Abcam, cat.no. ab194360, clone: EPR16893, 1:5000)
25. anti-HSP90 Antibody (Abcam, cat.no. ab87133, clone: polyclonal, 1:5000)
26. anti-HSP105 Antibody (Abcam, cat.no. ab109624, clone: EPR4576, 1:5000)
27. Goat Anti-Rabbit IgG (H+L) HRP (Horseradish peroxidase) Antibody (Affinity, cat.no. S0001, clone: Polyclonal, 1:2000)

Immunofluorescence:
28. anti-mouse CD11c Antibody (Abcam, cat.no. ab254183, clone: KB90, 1:100)
29. anti-mouse CD8 Antibody (Invitrogen, cat.no. 14-0081-82, clone: 53-6.7, 1:50)
30. anti-Ki67 Antibody(Abcam, cat.no. ab16667, clone: SP6, 1:100)
31. anti-Granzyme B Antibody (Abcam, cat.no. ab255598, clone: EPR22645-206, 1:200)
32. anti-Calreticulin Antibody, Alexa Fluor® 488 (Abcam, cat.no. ab196158, clone:EPR3924, 1:1000)
33. anti-HMGB1 Antibody, (Abcam, cat.no. ab79823, clone:EPR3507, 1:1000)
34. Goat anti-Mouse IgG (H+L) , Alexa Fluor Plus 647 (Invitrogen, cat.no. A48265, 1:1000)
35. Donkey Anti-Rat IgG (H+L) , Alexa Fluor® 555(Abcam, cat.no. ab150154, 1:300)
36. Goat Anti-Rabbit IgG (H+L), FITC (Affinity, cat.no. S0008, 1:300)

Validation

All antibodies were purchased from the supplier as noted above, and used without additional validation.
1. PE anti-mouse CD11c Antibody (Biolegend, cat.no.117308)
   https://www.biolegend.com/en-us/products/pe-anti-mouse-cd11c-antibody-1816
2. APC anti-mouse CD80 Antibody (Biolegend, cat.no.104713)
   https://www.biolegend.com/en-us/products/apc-anti-mouse-cd80-antibody-2340
3. APC/Cyanine7 anti-mouse CD86 Antibody (Biolegend, cat.no. 105030)
   https://www.biolegend.com/en-us/products/apc-cyanine7-anti-mouse-cd86-antibody-6554
4. PerCP/eFlour 710 anti-mouse MHC Class I (H-2Kd) Antibody (eBioscience, cat.no. 46-5957-82)
   https://www.thermofisher.cn/cn/zh/antibody/product/MHC-Class-I-H-2Kd-Antibody-clone-SF1-1-1-1-Monoclonal/46-5957-82
5. FITC anti-mouse CD11c Antibody (Biolegend, cat.no.117305)
   https://www.biolegend.com/en-us/products/fitc-anti-mouse-cd11c-antibody-1815

6. PerCP/Cyanine5.5 anti-mouse CD86 Antibody (Biolegend, cat.no. 105027)
https://www.biolegend.com/en-us/products/percp-cyanine5-5-anti-mouse-cd86-antibody-4276
7. PE/Cyanine7 anti-mouse CD80 Antibody (Biolegend, cat.no. 104733)
https://www.biolegend.com/en-us/products/pe-cyanine7-anti-mouse-cd80-antibody-9320
8. APC anti-mouse CD40 Antibody (Biolegend, cat.no. 124611)
https://www.biolegend.com/en-us/products/apc-anti-mouse-cd40-antibody-4984
9. APC anti-mouse CD4 Antibody (Biolegend, cat.no. 100411)
https://www.biolegend.com/en-us/products/apc-anti-mouse-cd4-antibody-245
10. PE anti-mouse CD3 Antibody (Biolegend, cat.no. 100206)
https://www.biolegend.com/en-us/products/pe-anti-mouse-cd3-antibody-47
11. Brilliant Violet 605 anti-mouse CD8a Antibody (Biolegend, cat.no. 100743)
https://www.biolegend.com/en-us/products/brilliant-violet-605-anti-mouse-cd8a-antibody-7636
12. PerCP/Cyanine5.5 anti-mouse IFN-γ Antibody (eBioscience, cat.no. 45-7311-82)
https://www.thermofisher.cn/cn/zh/antibody/product/IFN-gamma-Antibody-clone-XMG1-2-Monoclonal/45-7311-82
13. Alexa Flour647 anti-mouse granzyme B Antibody (Biolegend, cat.no. 515405)
https://www.biolegend.com/en-us/products/alexa-fluor-647-anti-human-mouse-granzyme-b-antibody-6067
14. eflour450 anti-mouse CD44 Antibody (eBioscience, cat.no. 48-0441-82)
https://www.thermofisher.cn/cn/zh/antibody/product/CD44-Antibody-clone-IM7-Monoclonal/48-0441-82
15. APC anti-mouse CD62L Antibody (Biolegend, cat.no. 104411)
https://www.biolegend.com/en-us/products/apc-anti-mouse-cd62l-antibody-381
16. FITC anti-mouse CD45 Antibody (Biolegend, cat.no. 157214)
https://www.biolegend.com/en-us/products/fitc-anti-mouse-cd45-antibody-21256
17. PE anti-human CD45 Antibody (Biolegend, cat.no. 304008)
https://www.biolegend.com/en-us/products/pe-anti-human-cd45-antibody-708
18. Brilliant Violet 605 anti-human CD3 Antibody (Biolegend, cat.no. 317322)
https://www.biolegend.com/en-us/products/brilliant-violet-605-anti-human-cd3-antibody-7666
19. PE- Cyanine7 anti-human CD4 Antibody (eBioscience, cat.no. 25-0049-42)
https://www.thermofisher.cn/cn/zh/antibody/product/CD4-Antibody-clone-RPA-T4-Monoclonal/25-0049-42
20. Alexa Flour700 anti-human CD8 Antibody (eBioscience, cat.no. 56-0088-42)
https://www.thermofisher.cn/cn/zh/antibody/product/CD8a-Antibody-clone-RPA-T8-Monoclonal/56-0088-42
21. Purified Rat Anti-Mouse CD16/CD32 (Mouse BD Fc Block™) (BD, cat.no. 553141)
https://www.bdbiosciences.com/zh-cn/products/reagents/flow-cytometry-reagents/research-reagents/single-color-antibodies-ruo/purified-rat-anti-mouse-cd16-cd32-mouse-bd-fc-block.553141
22. Ghost Dye UV450 (Tonbo, cat.no. 13-0863, 1:500)
https://tonbo-bioscience.myshopify.com/products/ghost-dye-violet-450
23. anti-GAPDH Antibody (Affinity, cat.no. AF7021, Human, Mouse, Rat)
https://www.affbiotech.cn/goods-6289-AF7021-GAPDH_Antibody.html
24. anti-HSP70 Antibody (Abcam, cat.no. ab194360, Mouse, Rat, Human)
https://www.abcam.cn/products/primary-antibodies/hsp70-antibody-epr16893-ab194360.html
25. anti-HSP90 Antibody (Abcam, cat.no. ab87133, Mouse, Rat, Human)
https://www.abcam.cn/products/primary-antibodies/hsp90-antibody-ab87133.html
26. anti-HSP105 Antibody (Abcam, cat.no. ab109624, Mouse, Rat, Human)
https://www.abcam.cn/products/primary-antibodies/hsp105hsp110-antibody-epr4576-ab109624.html
27. Goat Anti-Rabbit IgG (H+L) HRP (Horseradish peroxidase) Antibody (Affinity, cat.no. S0001)
https://www.affbiotech.cn/goods-6302-S0001-Goat_Anti_Rabbit_IgG_H_L_HRP.html
28. anti-mouse CD11c Antibody (Abcam, cat.no. ab254183, Mouse, Human)
https://www.abcam.cn/products/primary-antibodies/cd11c-antibody-kb90-ab254183.html
29. anti-mouse CD8 Antibody (Invitrogen, cat.no. 14-0081-82, Mouse, Rat, Human)
https://www.thermofisher.cn/cn/zh/antibody/product/CD8a-Antibody-clone-53-6-7-Monoclonal/14-0081-82
30. anti-Ki67 Antibody(Abcam, cat.no. ab16667, Mouse, Rat, Human)
https://www.abcam.cn/products/primary-antibodies/ki67-antibody-sp6-ab16667.html
31. anti-Granzyme B Antibody (Abcam, cat.no. ab255598, Mouse, Rat, Human)
https://www.abcam.cn/products/primary-antibodies/granzyme-b-antibody-epr22645-206-ab255598.html
32. anti-Calreticulin Antibody, Alexa Fluor® 488 (Abcam, cat.no. ab196158, Mouse, Rat, Human)
https://www.abcam.cn/products/primary-antibodies/alexa-fluor-488-calreticulin-antibody-epr3924-er-marker-ab196158.html
33. anti-HMGB1 Antibody, (Abcam, cat.no. ab79823, Mouse, Rat, Human)
https://www.abcam.cn/products/primary-antibodies/hmgb1-antibody-epr3507-ab79823.html
34. Goat anti-Mouse IgG (H+L) , Alexa Fluor Plus 647 (Invitrogen, cat.no. A48265)
https://www.thermofisher.cn/cn/zh/antibody/product/Goat-anti-Rat-IgG-H-L-Highly-Cross-Adsorbed-Secondary-Antibody-Polyclonal/A48265
35. Donkey Anti-Rat IgG (H+L) , Alexa Fluor® 555(Abcam, cat.no. ab150154)
https://www.abcam.cn/products/secondary-antibodies/donkey-rat-igg-hl-alexa-fluor-555-preadsorbed-ab150154.html
36. Goat Anti-Rabbit IgG (H+L), FITC (Affinity, cat.no. S0008)
https://www.affbiotech.cn/goods-13721-S0008-Goat_Anti_Rabbit_IgG_H_L_FITC_conjugated.html

Eukaryotic cell lines

Policy information about cell lines and Sex and Gender in Research

Cell line source(s)	4T1 murine breast cancer cells (catalog no. CL-0007), CT26 murine colorectal cancer cell line (catalog no. CL-0071), and LLC murine lung cancer cells (catalog no. CL-0140) were purchased from Procell Life Science&Technology Co.,Ltd (Wuhan, China). Luc-4T1 cells (catalog no. LZQ0016) and Luc-Pan02 murine pancreatic adenocarcinoma cells (catalog no. LZQ0041) were purchased from Zhong Qiao Xin Zhou Biotechnology Co.,Ltd (Shanghai, China).
Authentication	We used widely used cell lines from commercial sources, and these cells were authenticated by STR profiling.
Mycoplasma contamination	All the cell lines were tested termly and they were negative for mycoplasma contamination.
Commonly misidentified lines (See ICLAC register)	In this study, no commonly misidentified cell lines were used.

Animals and other research organisms

Policy information about studies involving animals; ARRIVE guidelines recommended for reporting animal research, and Sex and Gender in Research

Laboratory animals	BALB/c mice and C57BL/6 mice (6-8 weeks, female) were obtained from Vital River Laboratories. Ifng-IRES-Venus-AkaLuci mice were obtained from Shanghai Model Organisms Center. NOD.Cg-PrkdcscidIl2rgtm1Sug/ShiJic (NTG) mice (6-8 weeks, female) were purchased from SiPeiFu Biotechnology. CD4-KO and CD8-KO mice sourced from the Jackson Laboratory. All the model mice were raised in a standard environmentally controlled room (23 °C, with 55 ± 5% humidity and under a 12 h-12 h light-dark cycle).
Wild animals	This study did not involve wild animals.
Reporting on sex	This study used both male and female mice.
Field-collected samples	This study did not involve samples collected from the field.
Ethics oversight	This study was performed in strict accordance with the Regulations for the Care and Use of Laboratory Animals and Guideline for Ethical Review of Animal (China, GB/T 35892-2018). All animal experiments were reviewed and approved by the Animal Ethics Committee of the Institute of Process Engineering (approval ID: IPELLSC032101).

Note that full information on the approval of the study protocol must also be provided in the manuscript.

Flow Cytometry

Plots

Confirm that:

- [x] The axis labels state the marker and fluorochrome used (e.g. CD4-FITC).
- [x] The axis scales are clearly visible. Include numbers along axes only for bottom left plot of group (a 'group' is an analysis of identical markers).
- [x] All plots are contour plots with outliers or pseudocolor plots.
- [x] A numerical value for number of cells or percentage (with statistics) is provided.

Methodology

Sample preparation	4T1 cells were seeded onto 24-well plate and incubated with NPs and N-NPs (10 μg/mL) for 4 h, respectively. Subsequently, the amounts of NPs and N-NPs internalized in cells were determined using Backman CytoFLEX LX Flow Cytometer . Animals tissues were collected, ground up, sieved and stained with flourescence labelled antibodies. Then, the stained cells were washed by staining buffer and measured by Backman CytoFLEX LX Flow Cytometer and analyzed by associated software (version 2.3.1.22).
Instrument	The instrument for data collection is CytoFLEX LX (BECKMAN COULTER).
Software	The flow cytometry data were analyzed by CytExpert(version 2.3.1.22).
Cell population abundance	For the in vivo studies, 1/3 of the collected tissue samples and 100 μL of peripheral blood were used for per measurement.
Gating strategy	Generally, cells was first gated on FSC-A/SSC-A. Singlet cells were usually gated using FSC-H and FSC-A.

- [x] Tick this box to confirm that a figure exemplifying the gating strategy is provided in the Supplementary Information.
